# 1,2,3-triazole and chiral Schiff base hybrids as potential anticancer agents: DFT, molecular docking and ADME studies

**DOI:** 10.1038/s41598-024-57689-5

**Published:** 2024-03-23

**Authors:** Yonas Belay, Alfred Muller, Fanikie S. Mokoena, Adedapo S. Adeyinka, Lesetja R. Motadi, Abel K. Oyebamiji

**Affiliations:** 1 Department of Chemical Sciences, University of Johannesburg, P.O. Box 524, Auckland Park, 2006 South Africa; 2 Department of Biochemistry, University of Johannesburg, P.O. Box 524, Auckland Park, 2006 South Africa; 3 Industrial Chemistry Programme, Bowen University, PMB 284, Iwo, Osun State Nigeria

**Keywords:** Cancer, Hybrid drugs, 1,2,3-triazole, Chiral Schiff bases, Molecular docking, ADME, Cancer, Drug discovery, Chemistry

## Abstract

A series of novel 1,2,3-triazole and chiral Schiff base hybrids **2**–**6** were synthesized by Schiff base condensation reaction from pre-prepared parent component of the hybrids (1,2,3-triazole **1)** and series of primary chiral amines and their chemical structure were confirmed using NMR and FTIR spectroscopies, and CHN elemental analysis. Compounds **1**–**6** were evaluated for their anticancer activity against two cancer PC3 (prostate) and A375 (skin) and MRC-5 (healthy) cell lines by Almar Blue assay method. The compounds exhibited significant cytotoxicity against the tested cancer cell lines. Among the tested compounds **3** and **6** showed very good activity for the inhibition of the cancer cell lines and low toxicity for the healthy cell lines. All the compounds exhibited high binding affinity for Androgen receptor modulators (PDB ID: 5t8e) and Human MIA (PDB ID: 1i1j) inhibitors compared to the reference anticancer drug (cisplatin). Structure activity relationships (SARs) of the tested compounds is in good agreement with DFT and molecular docking studies. The compounds exhibited desirable physicochemical properties for drug likeness.

## Introduction

Cancer is a complex disease, and it ranks as the second leading cause of mortality worldwide^1,2^. It was estimated that 19.3 million new cases of cancer and almost 10.0 million cancer-related deaths were reported in 2020^3^. According to global demographic trends, 420 million new cancer cases are expected annually by 2025^4^. The current available drugs that have been employed for cancer treatment are not effective due to lack of efficacy and poor selectivity, the latter of which could lead to adverse side effects^5^. In addition, the emergence of drug resistance has hampered the effectiveness of these drugs in the clinic^6^. Thus, there is an urgent need to develop effective, safe, and selective anticancer agents with enhanced properties that could overcome current limitations in chemotherapy treatment. A combination of two or more pharmacophores or synthesis of hybrid molecules constituting two or more bioactive entities could be a viable solution to fight drug resistance in cancer cells^7–10^. Hybrid drugs can extend the spectrum of biological activity, enhance the potency, overcome drug resistance, reduce side effects, and improve pharmacokinetic, pharmacodynamic as well as physicochemical profiles^11–15^. Literature reports indicated that several hybrids have been synthesized and are under different phase clinical trials for the treatment of various diseases including those caused by drug-resistant organisms, revealing hybridization is a useful strategy to develop novel anticancer drugs^16^.

**Figure 1:**
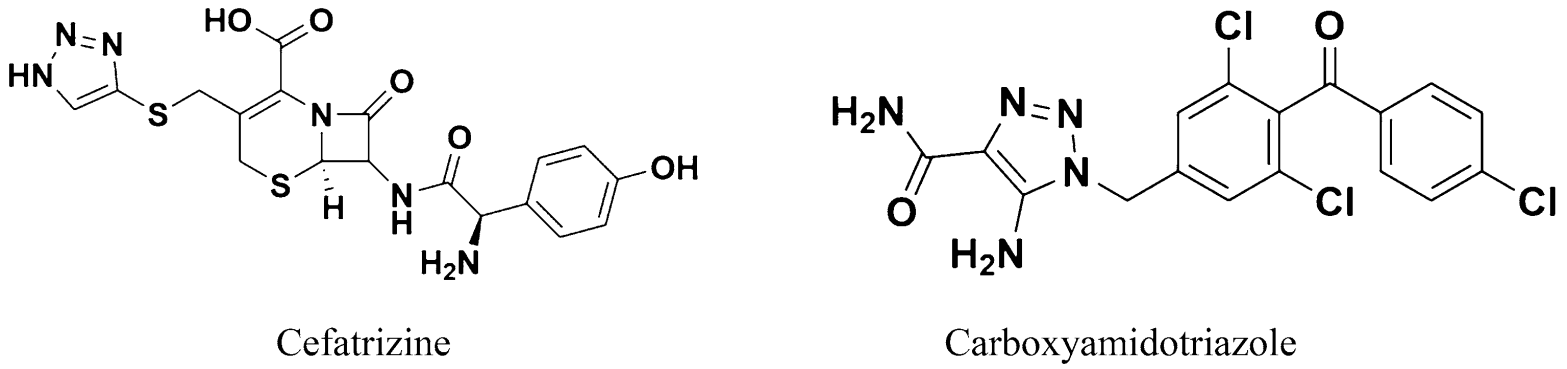
Reported 1,2,3-triazole containing compounds which are under clinical evaluation for cancer treatment.

1,2,3-Triazoles are one of the most important classes of nitrogen-containing heterocycles and can form various non-covalent interactions such as hydrophobic interactions, hydrogen bonds, van der Waals forces and dipole–dipole bonds with various enzymes, proteins, and receptors^17,18^. Therefore, triazole derivatives have attracted considerable attention due to their chemotherapeutic values such as antibacterial^11,19,20^, antimalarial^21,22^, antiviral^23^, antifungal^24^, antitubercular^25,26^, and anticancer activities^27–31^. Reported examples of 1,2,3-triazole-containing compounds that exhibited anticancer activity are Cefatrizine derivative, carboxyamido-triazoles, azido-*β*-lactam, amprenavir derivative, 1,2,3-triazole-dithiocarbamate-urea hybrid and N-((1-benzyl-1*H*-1,2,3-triazole-4-yl)methyl)arylamid derivative. Cefatrizine and Carboxyamido-triazoles (Fig. 1) have already been used in clinics or are under clinical evaluation for cancer treatment, revealing their potential as putative anticancer drugs^32^.

Schiff bases are organic compounds formed by the condensation reaction of aldehydes or ketones with primary amines^34^. The presence of a lone pair of electrons of the nitrogen atoms’ *sp*^*2*^ hybridized orbital in the azomethine group is critical for their chemical and biological activities^35^ that render these as viable candidates for the anticancer properties of Schiff bases^36–38^ and their metal complexes^39–41^.

Chiral drugs are at the forefront of pharmaceutical drug research as introduction of chirality not only enforces stereo-selective specific drug interaction but also promotes the formation of active compounds with therapeutic benefits as most of the biotargets viz., DNA (the primary intracellular target) is chiral in nature^42^. Being inherently chiral, the double-stranded helical DNA can interact with chiral substrate resulting in different enantiomeric structures in a stereospecific way leading to different DNA-substrate adduct profiles^43^. On the other hand, the presence of planar molecules substituted with free functional groups like alcoholic, phenolate, amine, oxime, etc. might have a superior chemical binding profile with DNA molecules^43^. Inspired by the reported anticancer properties of 1,2,3-triazole, Schiff bases and chiral compounds and as a continuation of our research on the synthesis of 1,2,3-triazole hybrid compounds for medicinal applications^44^ herein, we report the synthesis of 1,2,3-triazole and chiral Schiff base hybrids and evaluation of their anticancer activities, DFT, molecular docking and druglikeness studies.

## Results and discussions

**Figure 2:**
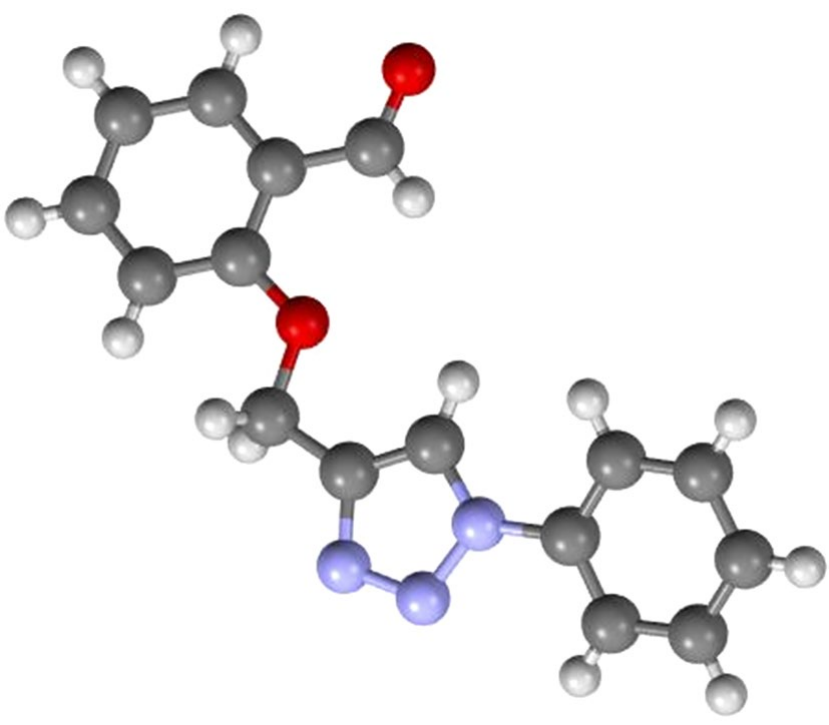
Crystal structure of compound **1**.

The one component of the hybrids, 1,2,3-triazole **1** was Previously synthesized through multistep synthesis by copper catalyzed click chemistry reaction of preprepared 2-(prop-2-yn-1-yloxy) benzaldehyde with azidobenzene. The structure of the compound was confirmed by ^1^H NMR spectroscopy. The disappearance of the signal for the terminal alkyne proton from 2-(prop-2-yn-1-yloxy) benzaldehyde at 2.55 ppm and appearance of the diagnostic low field singlet signal for the triazole ring proton at 8.08 ppm established the structure of 2-((1-phenyl-1H-1,2,3-triazol-4-yl)methoxy)benzaldehyde (**1**) (Fig. S1)^44^. Other characterization techniques such as FTIR, mass spectrometry and CHN elemental analysis were performed which established the formation of **1** (see experimental procedure). Compound **1** was exposed to different solvents for obtaining single crystals suitable for single crystal X-ray diffraction. Single crystals of **1** was obtained after one week from ethanol by slow evaporation. Single crystal analysis (Fig. 2) revealed that **1** crystallized in triclinic space group *Pī*. Details of the X-ray crystallographic data for the compound are given in Table 1 and rest of the structural parameters (bond lengths/angles and hydrogen parameters) are provided in the supplementary documents (Table S1).

**Table 1:** Crystal data and structure refinement for compound **1**.

Identification code	CCDC 2297996
Empirical formula	C_34_ H_28_ N_4_ O_4_
Formula weight	556.60
Temperature	173 (2) K
Wavelength	0.71073 Å
Crystal system	Triclinic
Space group	P$\overline{1}$
a/Å	4.4158 (14)
b/Å	15.086 (5)
c/Å	19.788 (7)
α/°	83.221 (11)
β/°	90
γ/°	90
Volume	1309.0(8) Å^3^
Z	2
Density (calculated)	1.412 Mg/m^3^
Absorption coefficient	0.094 mm^−1^
F(000)	584
Crystal size	0.300 × 0.110 × 0.100 mm^3^
Theta range for data collection	1.036 to 28.641°
Index ranges	− 5 ≤ h ≤ 5, − 20 ≤ k ≤ 19, − 26 ≤ l ≤ 26
Reflections collected	20,610
Independent reflections	6372 [R(int) = 0.1454]
Completeness to theta = 25.242°	98.9%
Refinement method	Full-matrix least-squares on F^2^
Data/restraints/parameters	6372/0/379
Goodness-of-fit on F^2^	0.869
Final R indices [I > 2sigma (I)]	R1 = 0.0734, wR2 = 0.1556
R indices (all data)	R1 = 0.2329, wR2 = 0.2259
Extinction coefficient	n/a
Largest diff. peak and hole	0.252 and − 0.296 e.Å^−3^

### Synthesis of hybrids of 1,2,3-triazole and chiral Schiff bases (2–6)

**Figure 3:**
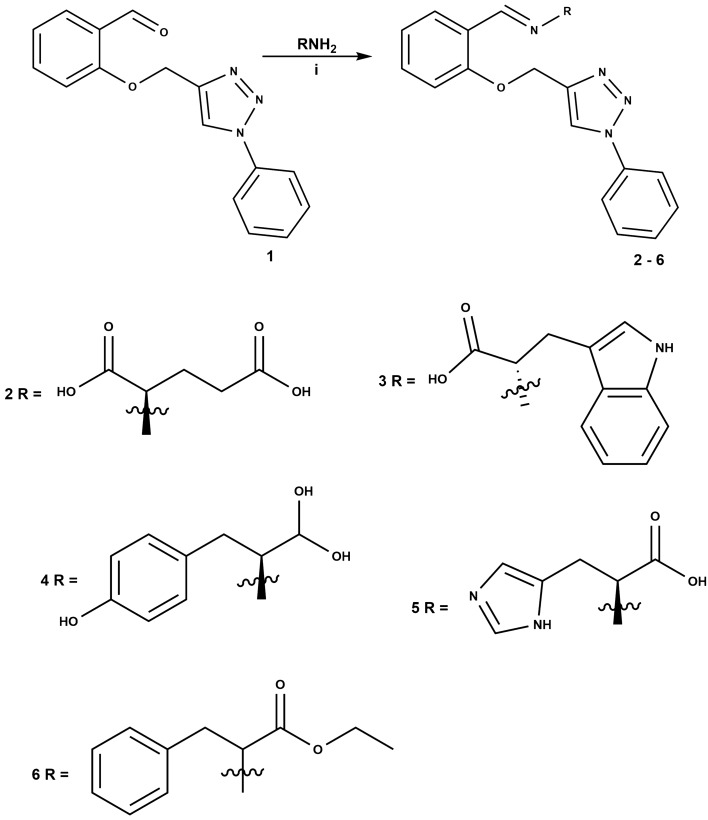
Synthesis of the hybrids of 1,2,3-triazole and chiral Schiff bases. (i) NaOH, MeOH, 80 °C, 1 h.

The reaction for the synthesis of the hybrid compounds is shown in Fig. 3. The reaction was performed by Schiff base condensation of the preprepared triazole **1** with primary chiral amines (*D*-glutamic acid, *L*-tryptophan, *L*-tyrosine and *L*-histidine) and an enantiomer Phenylalanine ethyl ester hydrochloride in the presence of sodium hydroxide as a base and provided the proposed hybrids of 1,2,3-triazole and chiral Schiff bases (**2**–**6**). The structures of the compounds were characterized using NMR and FTIR spectroscopy (see supplementary material Figs. S1–S14) as well as CHN elemental analysis.

The ^1^H NMR spectra of compounds (**2**–**6**) showed characteristic signals of the protons corresponding to the imine moiety between 9.02 and 8.06 ppm and the 1,2,3-triazole group between 8.92 and 7.92 ppm. The appearance of characteristic high field singlet signal between 5.55 and 2.68 ppm and between 5.26 and 2.63 ppm, respectively was attributed to the methine and methylene protons of the chiral amino acids. The appearance of characteristic high field singlet signal between 5.44 and 5.21 ppm was attributed to the methylene protons attached to the phenolic and 1,2,3-triazole groups. Moreover, the appearance of characteristic low field broad singlet signals in compounds **2** and **6** between 10.41 and 10.34 ppm and at 10.42 ppm, respectively was assigned to the hydroxyl protons of the chiral amino acids (*D*-glutamic acid and phenyl alanine). The broad singlet signal at 10.68 ppm in compound **3** is evident for the NH proton of the chiral amino acid (*L*-tryptophan). The absence of characteristic low field broad singlet signal in compounds **3**, **4**, and **5** is due to the deprotonation of the hydroxyl protons of the chiral amino acids by sodium hydroxide. In ^13^C NMR spectra, the presence of characteristic signals between 160.4 and 157.0 ppm correspond to the imine carbon (C=N). The presence of characteristic signals between 144.3 and 143.3 ppm for N–*C*=CH and between 120.3 and 120.1 ppm for N–C=*C*H, correspond to the 1,2,3-triazole carbons. The carbonyl carbon is evident between 192.1 and 174.8 ppm. The characteristic signals between 98.6 and 59.7 ppm for N–*C*H–CH_2_ and between 55.5 and 30.1 ppm for N–CH–*C*H_2,_ respectively correspond to the methine and methylene carbons of the chiral amino acids. The characteristic signal for the methylene carbon (*C*H_2_OAr) is attributed between 61.9 and 56.6 ppm. The molecular structures of compounds **2**–**6** were also characterized by FTIR spectroscopy (Fig. S14). The FTIR absorption spectra of compounds showed characteristic bands of the imine moiety between 1595 and 1598 cm^−1^ corresponding to the stretching of C=N bond. In compound **2**, the broad absorption band at *ca*. 3479 cm^−1^ and the strong absorption peak at 1667 cm^−1^ were attributed to the υ (OH stretch) and υ (C=O stretch) of the glutamic acid, respectively. In compound **6**, the broad absorption band at *ca*. 3353 cm^−1^ was attributed to the υ (OH stretch) of the phenyl alanine. The absence of characteristic broad absorption band for υ (OH stretch) in the FTIR spectra of compounds **3**, **4**, and **5** is evident for the deprotonation of the hydroxyl protons of the chiral amino acids by sodium hydroxide. Summary of ^1^H and ^13^C chemical shift of azomethine moiety, IR absorption of C=N and CHN elemental analysis results for compounds **2**–**6** is shown in Table 2.

**Table 2:** Synthesized hybrids of 1,2,3-triazole and chiral Schiff bases (**2**–**6**), ^1^H and ^13^C NMR chemical shifts of azomethine moiety, IR absorption of C = N and their CHN elemental analysis.

R-NH_2_	Product	^1^H NMR *H*-C=N (ppm)	^13^C NMR C=N (ppm)	IR υ (cm^−1^) C=N	CHN: anal. cald (found) %		
					C	H	N
	**2**	9.00	160.4	1595	61.76 (61.08)	4.94 (5.32)	13.72 (13.14)
	**3**	8.89	157.0	1595	66.52 (66.13)	4.55 (4.96)	14.37 (13.98)
	**4**	8.07	157.3	1598	61.73 (61.34)	4.14 (4.52)	11.52 (10.85)
	**5**	8.06	157.1	1584	60.27 (59.61)	4.37 (4.79)	19.17 (18.58)
	**6**	9.02	160.3	1578	70.41 (69.75)	5.20 (5.59)	13.14 (12.52)

### Anticancer study

The previously synthesized parent component of the hybrids (1,2,3-triazole) **1** and hybrids of **2**–**6** were evaluated for their anticancer activities against PC3 (prostate) and A375 (skin) cancer and MRC5 (healthy lung) cell lines with various concentrations of 100, 75, 50, 25, 15 and 5 μg/mL. The test results for IC_50_ values are presented in Table 3. Cisplatin was used as standard, and it showed an IC_50_ value of 30.11 μg/mL for the cancer cells and an IC_50_ value of 60.34 μg/mL for MRC5 (normal) cell. All the compounds showed lower activity for the inhibition of the PC3 cancer cell lines with IC_50_ value in the range of 40.46–75.05 μg/mL compared to the standard cisplatin with IC_50_ value of 30.11 μg/mL. Among the tested compounds **3** and **6** exhibited very good activity with IC_50_ values of 40.46 and 45.00 μg/mL, respectively. Compounds **2**, **4** and **5** showed moderate activity and **1** showed lower activity with IC_50_ value of 75.05 μg/mL for the proliferation of the PC3 cancer cell lines. For the inhibition of A375 cancer cell lines, most of the compounds exhibited anticancer activity with IC_50_ values in the range of 21.86–40.37 μg/mL comparable to the standard cisplatin with IC_50_ value of 30.11 μg/mL. Compounds **3**–**6** showed better activity than the standard cisplatin with IC_50_ values in the range of 21.86–28.94 μg/mL, while **1** and **2** exhibited very good activity at the concentration of 36.12–40.37 μg/mL. Compounds **1**–**6** were also evaluated for their cytotoxicity against MRC5 normal cells. Surprisingly, all the compounds exhibited less toxicity with IC_50_ values in the range of 76.90–93.07 μg/mL better than the standard cisplatin which showed high toxicity at a concentration of 60.34 μg/mL.

**Table 3:** IC_50_ values of compounds **1**–**6** screened for cytotoxicity against cancer (PC3 and A375) and MRC5 (normal) cell lines^***a***^.

Compound	Cytotoxicity (IC_50_, µg/mL)		
	PC3	A375	MRC5
**1**	75.05 ± 1.82	40.37 ± 1.03	76.90 ± 1.87
**2**	60.59 ± 1.42	36.12 ± 0.87	85.33 ± 2.27
**3**	40.46 ± 1.06	21.86 ± 0.52	86.40 ± 2.64
**4**	55.69 ± 1.28	24.83 ± 0.61	88.40 ± 2.38
**5**	60.53 ± 1.34	28.94 ± 0.65	79.42 ± 2.71
**6**	45.00 ± 1.12	24.18 ± 0.57	93.07 ± 2.42
Cisplatin	30.11 ± 0.76	30.11 ± 0.69	60.34 ± 1.23

^a^IC_50_ values are expressed as mean ± SD of three independent experiments.

The test results for cytotoxicity and IC_50_ values of compounds **1**–**6** treated against PC3 cancer cell lines at different concentrations 5, 15, 25, 50, 75 and 100 μg/mL is presented in Table S2. The cytotoxicity is concentration dependent and all the compounds except for **3** and **6** did not show any significant potency for the inhibition of the cancer cells in the concentration range of 5–50 μg/mL. All the compounds except for **3** did exhibit any significant activity in the concentration range of 5–25 μg/mL for the inhibition of A375 cancer cell lines (Table S3). Based on IC_50_ values of tested compounds, the order of cytotoxicity is **3** > **6** > **4** > **5** > **2** > **1** and this revealed that hybridisation of **1** with the series of chiral amines played a vital role for the activity. Among the hybrids, the high activity of **3** and **6** could be due to the presence of heterocyclic and lipophilic substituents, respectively, on the Schiff base component of the hybrids. The experiment was repeated three times, with the finding reported as mean ± SD (Figs. S15 and S16).

**Figure 4:**
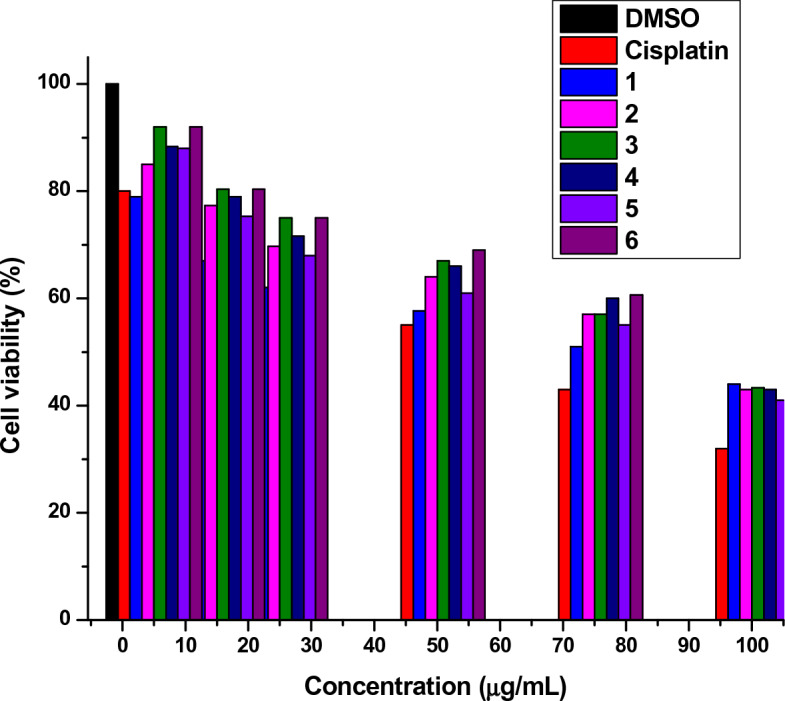
Cell viability (%) of MRC5 normal cell lines treated with various concentrations (5–100 μg/mL) of compounds **1**–**6** and cisplatin vs cells treated in DMSO (0.1%).

For a compound to be a potential anticancer drug candidate it must be nontoxic to healthy cell lines. Compounds **1**–**6** were screened for their toxicity against MRC5 normal cell lines at various concentrations (5–100 μg/mL) and their test results are depicted in Fig. 4. All the compounds were found to be nontoxic and exhibited cell viability (%) 51–92, at the concentration range of 5–75 μg/mL better than the standard cisplatin, which showed cell viability (%) 55–80, at the concentration range of 5–50 μg/mL. Therefore, the compounds could be viable potential candidates for the development of new anticancer drugs.

The selectivity index (SI) shown in Table 4, was calculated as the ratio of the IC_50_ for the normal cell line (MRC5) to the IC_50_ for a respective cancerous cell line. Higher values of SI indicate greater anticancer specificity and the compounds displaying SI values higher than 3 were considered to be highly selective^45^. Some of the compounds not only had high cytotoxic activity against cancer cells but also displayed low toxicity against normal (MRC5) cells and their SI values were higher than 3.5. The SI values of compound **3** and **6** in A375 cancer cells were 3.95 and 3.85, respectively. The two compounds have high cytotoxicity to the cancer cells and low cytotoxicity to healthy cell lines. Compounds **3** and **6** were selected as potent compounds for further investigation using computational and molecular docking studies.

**Table 4:** The calculated values of the selectivity index (SI) for compounds **1**–**6**.

Compound	SI	
	PC3	A375
**1**	1.02	1.90
**2**	1.41	2.36
**3**	2.13	3.95
**4**	1.59	3.56
**5**	1.31	2.74
**6**	2.07	3.85

### DFT study and chemical reactivity parameters

**Figure 5:**
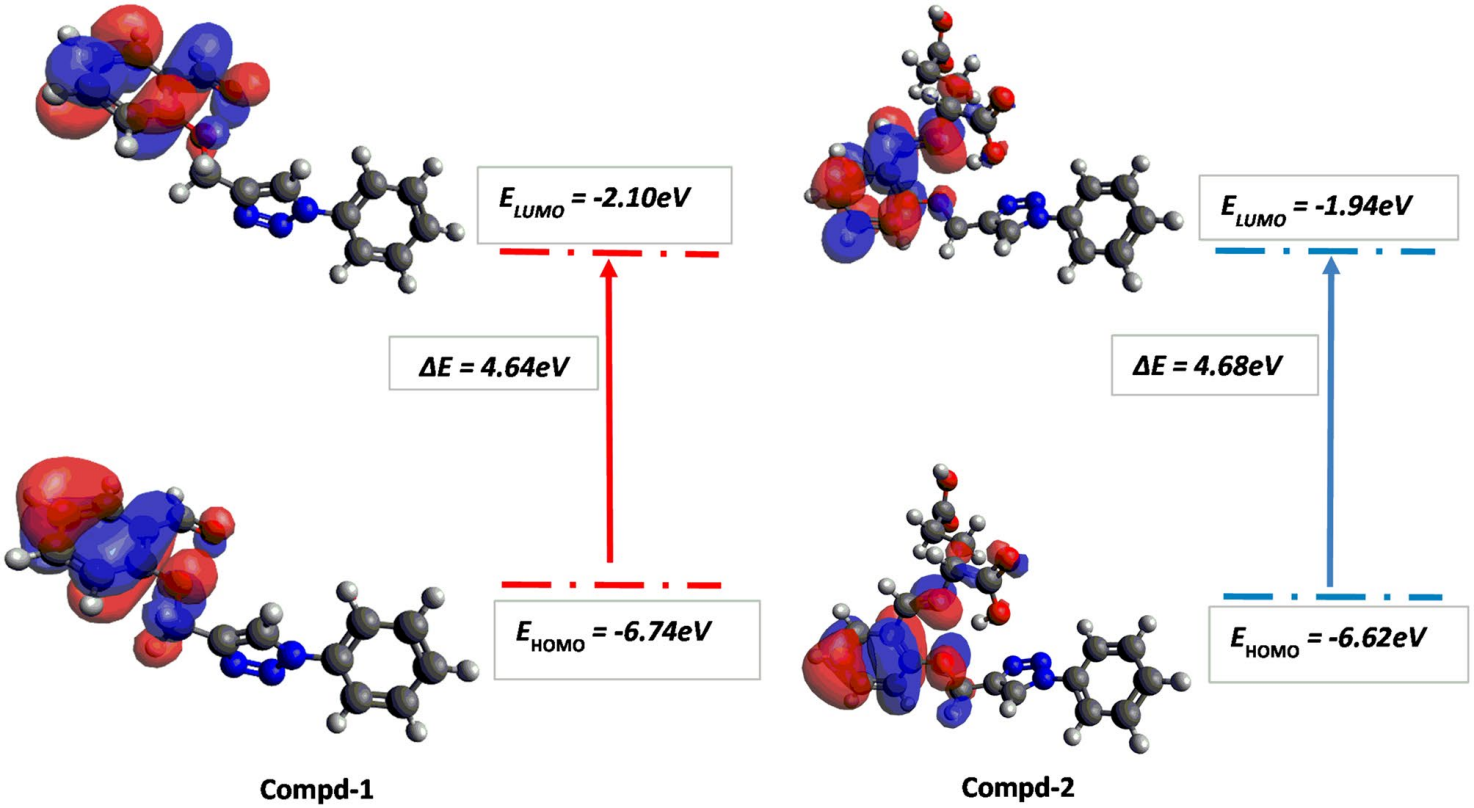
Frontier molecular orbital diagram and energy values for compounds **1** and **2**.

**Figure 6:**
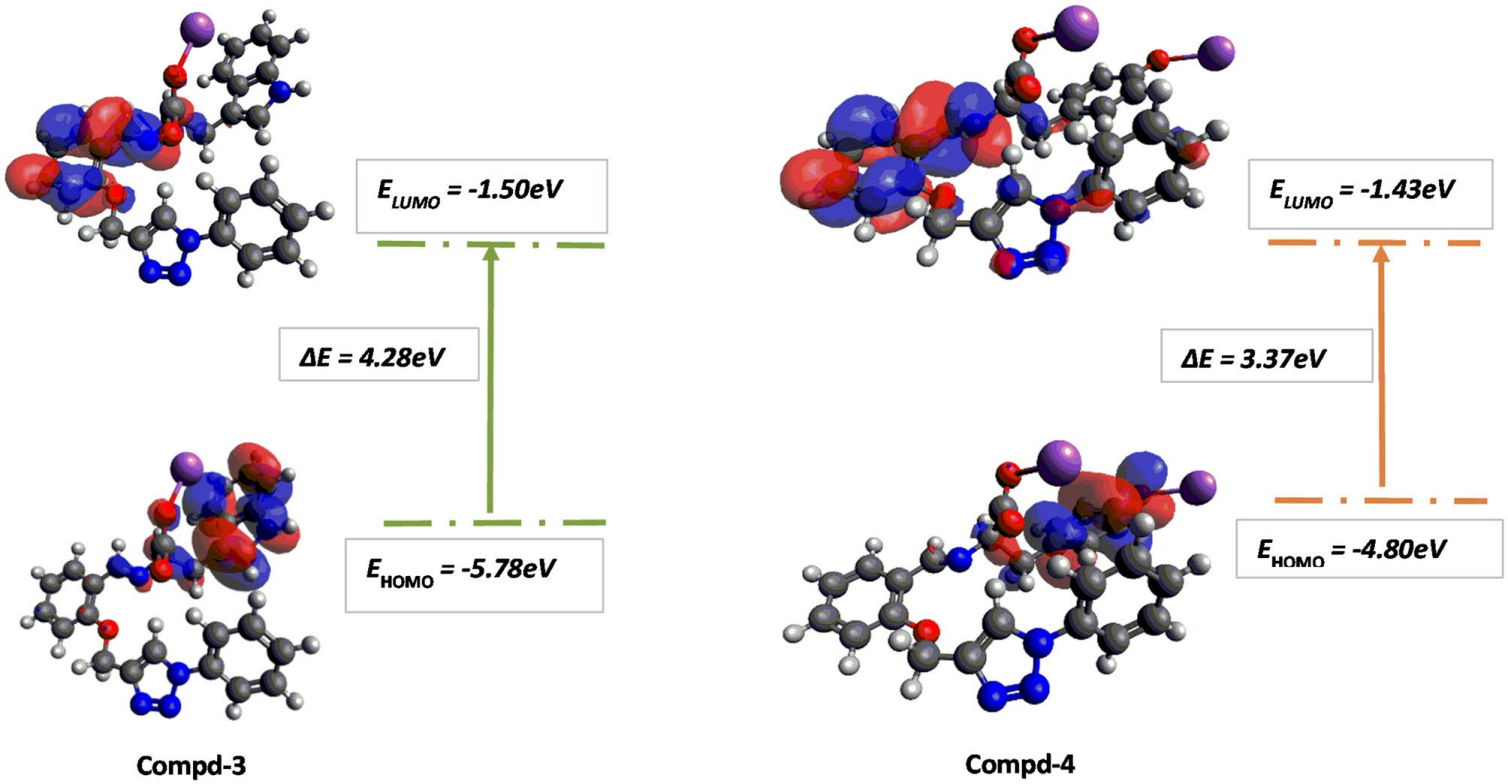
Frontier molecular orbital diagram and energy values for compounds **3** and **4**.

**Figure 7:**
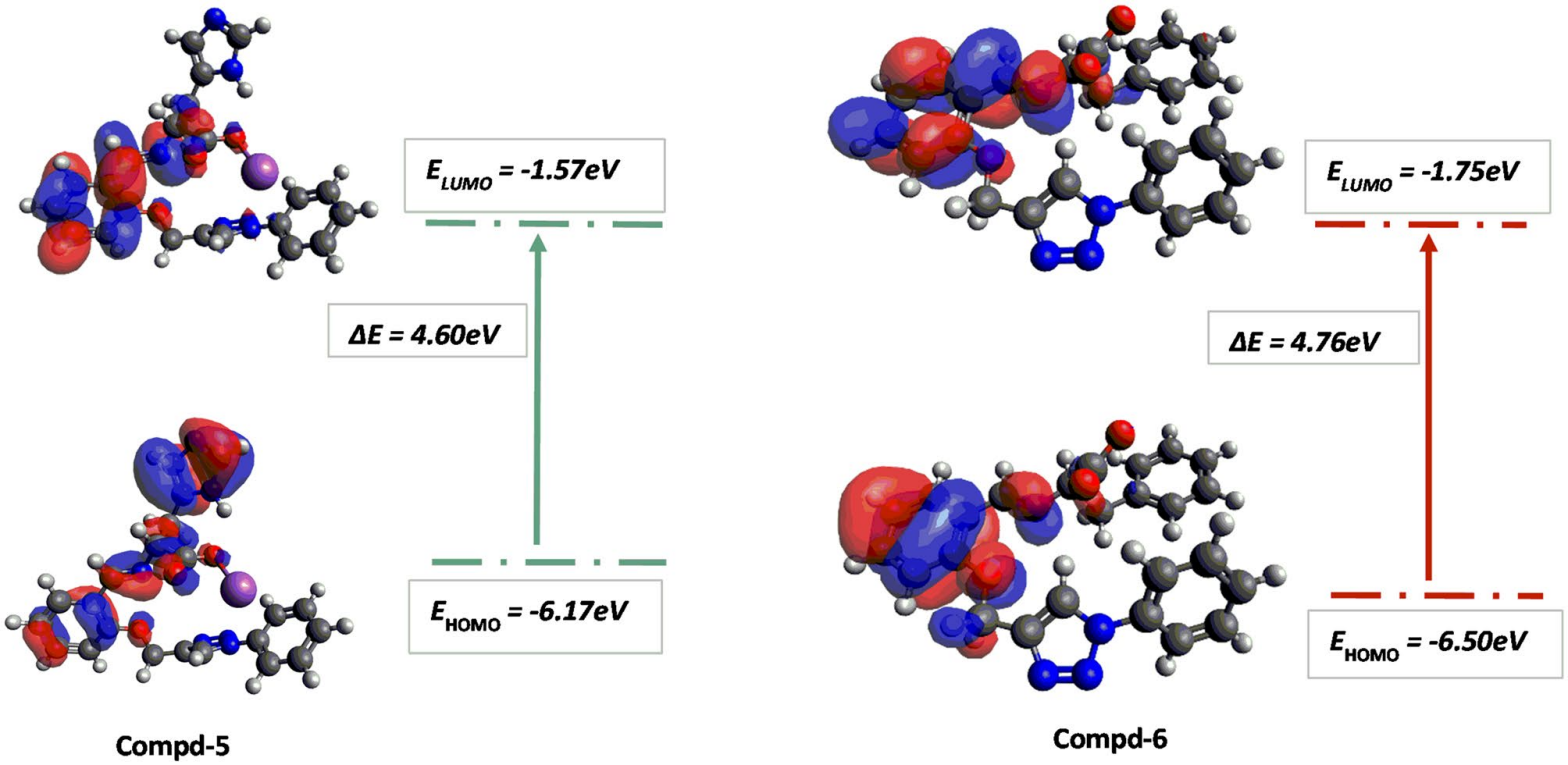
Frontier molecular orbital diagram and energy values for compounds **5** and **6**.

DFT studies were carried out on all the compounds. Their optimized ground state geometries was obtained at the B3LYP-GD3/6–311 +  + G(d,p) level of theory using Gaussian16 Rev B.01 software. The frontier molecular orbitals, HOMO and LUMO were studied as well as the energy gap between the HOMO–LUMO orbitals which is indicative of the stability and reactivity of these compounds. The HOMO is a region in which electrons can be transferred to unoccupied orbitals, while LUMO is an electron-accepting spot. HOMO was found as bonding orbitals that are dispersed throughout the molecule. Several studies have shown that a lower ΔE HOMO–LUMO corresponds to more bioactivity of a molecule, and this has been used to explain the relative bioactivities of medicinal compounds^46^. Figures 5, 6 and 7 show the HOMO and LUMO of compounds **1**, **2**, **3**, **4**, **5** and **6** as well as the energy gap between these frontier orbitals. In all the compounds the HOMO and LUMO are only pronounced on the Schiff base component of the hybrid compound. As shown in Fig. 5, for compound **1** its HOMO and LUMO are evenly distributed over the whole molecule, but the HOMO is more delocalized over the component atoms when compared to its LUMO. In contrast, the main contributions to the HOMO and LUMO of compound **2** are the atoms in its benzene ring and imine moiety. The ΔE HOMO–LUMO for compound **2** is slightly greater than **1** and this is due to the replacement of –C (H)=O group from compound **1** by –C (H)=N group in compound **2**.

Figure 6 shows the HOMO and LUMO for compounds **3** and **4**. In both compounds the LUMO is pronounced on the aromatic group from the parent compound and the imine moiety. The HOMO for **3** is distributed on the imine moiety and the tryptophan component, however, in compound **4** the HOMO is delocalized only on the aromatic group of the tyrosine component. For compound **4**, the ΔE HOMO–LUMO is lower than compound **3** and this is due to the replacement of the tryptophan subunit linked to a sodium ion with a tyrosine subunit linked to two sodium ions.

As shown in Fig. 7, for compound **6** the HOMO and LUMO are evenly distributed on the aromatic group of the parent component and the imine moiety. For compound **5** the HOMO is delocalized over the whole aromatic groups of the Schiff base components (aldehyde and amine sources) and the LUMO is only pronounced on the aromatic group of the parent component and the imine moiety. For compound **5**, the ΔE HOMO–LUMO is lower than compound **6** and this is due to the replacement of the phenyl alanine subunit with a histidine subunit linked to a sodium ion.

The ΔE HOMO–LUMO is 4.76, 4.68, 4.64, 4.60, 4.28 and 3.37 eV for compounds **6**, **2**, **1**, **5**, **3** and **4** respectively. So, assuming compound **1** is the parent compound, it is observed that the energy gap in compounds **6** and **2** increase slightly due to the replacement of the aldehydic moiety from compound **1** by the imine moiety linked to glutamic acid and phenyl alanine subunit, respectively. For compound **5** decreases slightly due to the replacement of the aldehydic moiety from compound **1** by the imine moiety linked to histidine subunit. The energy gap in compounds **3** and **4** decreases significantly due to the replacement of the aldehyde subunit with an imine moiety linked to tryptophan and tyrosine subunit, respectively.

**Figure 8:**
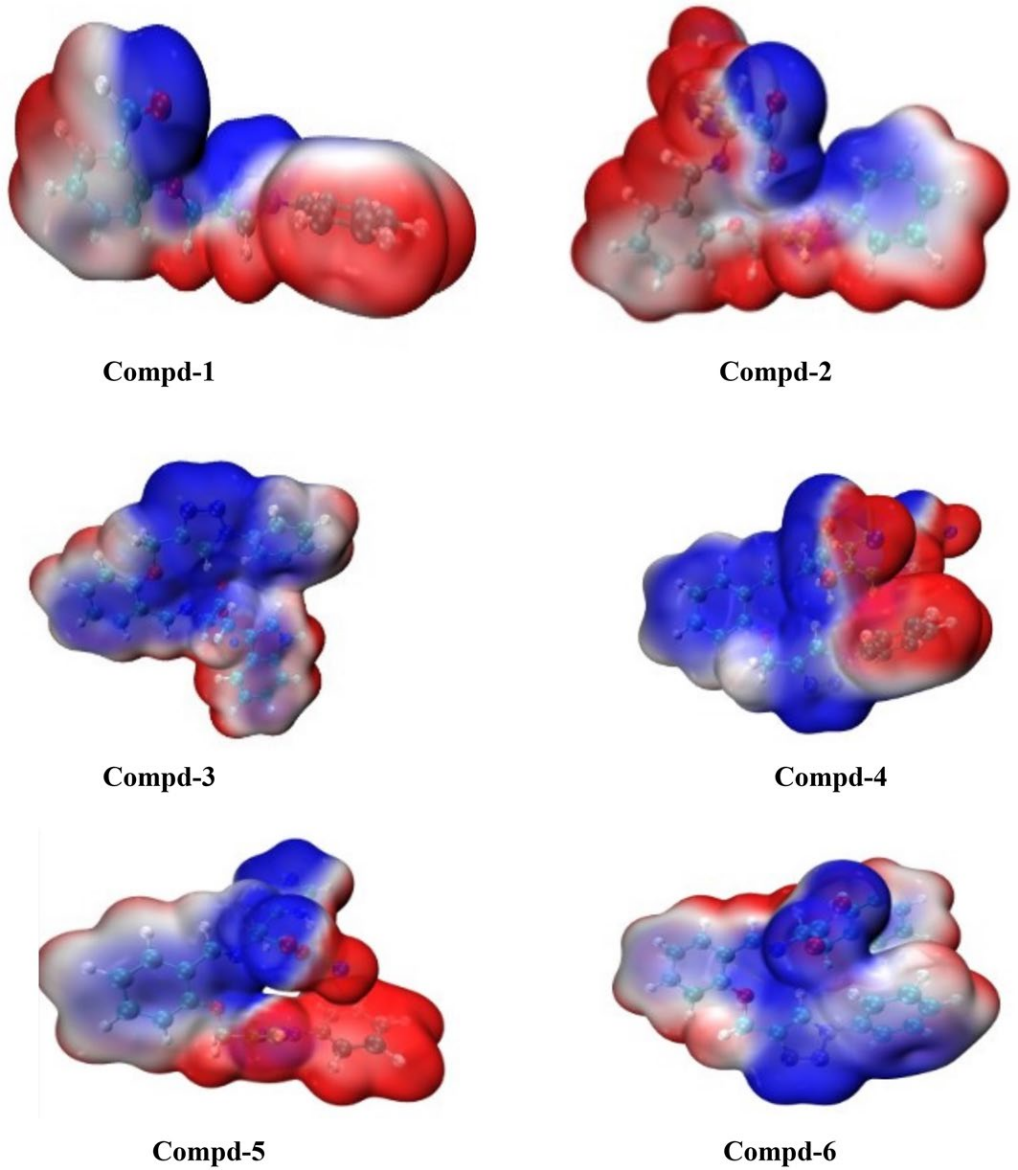
Molecular electrostatic potential maps of compounds **1**, **2**, **3**, **4**, **5** and **6**.

The Molecular Electrostatic potential (MEP) for compounds **1**, **2**, **3**, **4**, **5**, and **6** is shown in Fig. 8. MEP indicates the net electrostatic effect exerted at a point in space by the total charge distribution over a molecule. It can be used to study the reactivity of molecules towards electrophilic and nucleophilic reagents as well as their drug-receptor interactions. The different colours on the surfaces (Fig. 8) are indicative of their electrostatic potential values; it increases in the order red < orange < green < blue, where the higher electrostatic potential negative (red) regions of the MEP map are related to electrophilic attack reactivity, whereas the positive (blue) regions are related to nucleophilic attack reactivity, neutral region is represented by green colour. For compound **1**, the positive (blue) regions are localized in the oxygen atoms of the carbonyl and ether and on nitrogen atoms of the 1,2,3-triazole ring. The negative (red) regions are found on the aromatic subunit linked to the 1,2,3-triazole component. For compound **2**, the negative (red) regions are distributed across most of the components of the molecule and the positive (blue) regions are localized on the two oxygen atoms of the carboxylate and partially on the aromatic subunit linked to the 1,2,3-triazole component. For compounds **3** and **6**, the positive (blue) regions are diffused across the whole components of the molecules and the few negative (red) regions are found on the aromatic hydrogen atoms. For compound **4**, the positive (blue) regions are found in most of the components of the molecule. The negative (red) regions are localized on the two sodium ions linked to the tyrosine subunit and on the aromatic subunit linked to the 1,2,3-triazole component. For compound **5**, the positive (blue) regions are distributed across the Schiff base component of the molecule and the negative (red) regions are localized on the 1,2,3-triazole, aromatic subunit linked to the triazole and on the sodium ion of the carboxylate moiety of histidine subunit. Therefore, the presence of positive (blue) and negative (red) regions on the molecules are evidence for the potential bioactivity of the compounds that could interact through its electrophilic and nucleophilic sites.

### Molecular docking analysis

**Figure 9:**
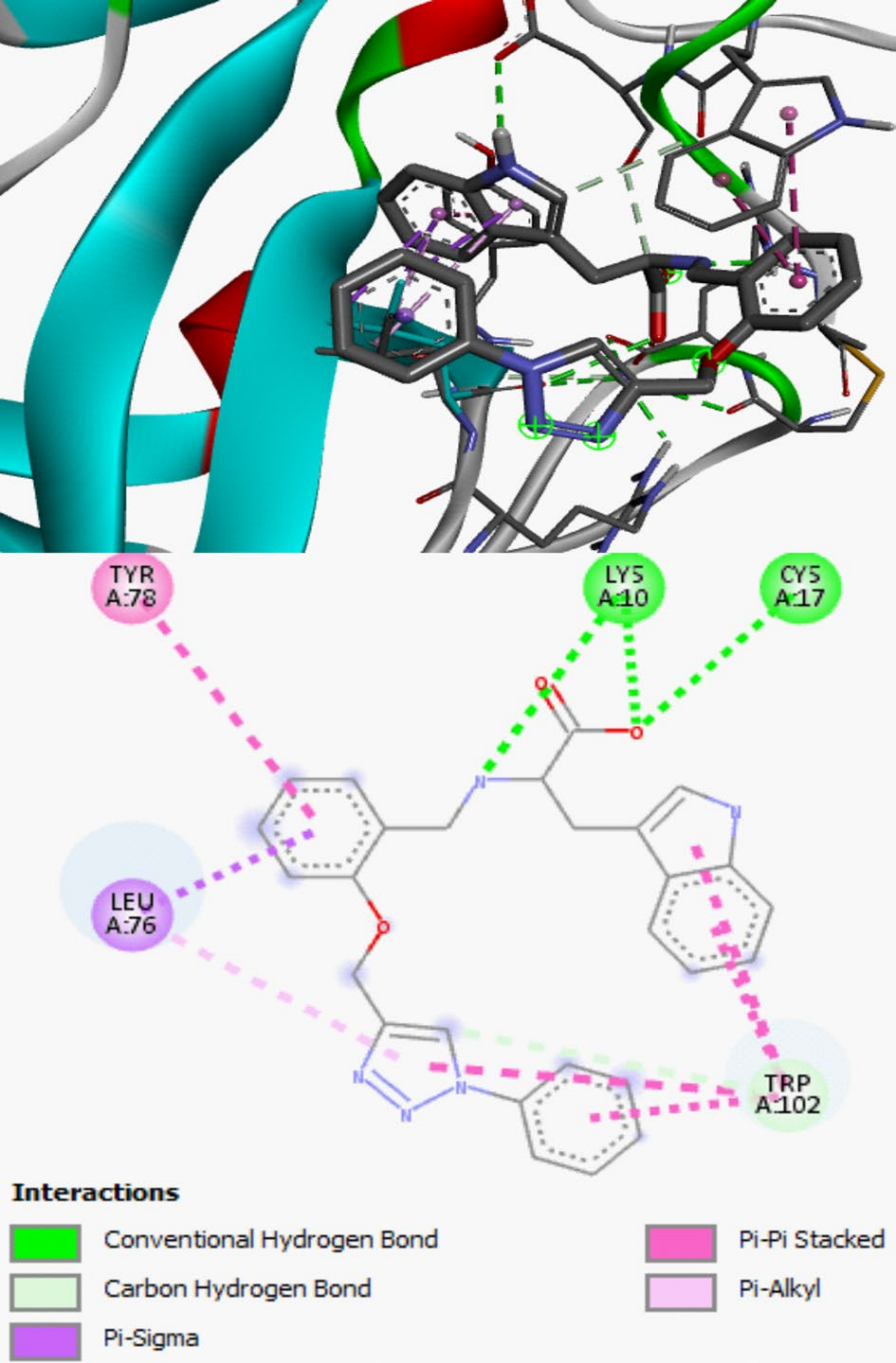
3D and 2D structure of compound **3** in the active site of human MIA.

**Figure 10:**
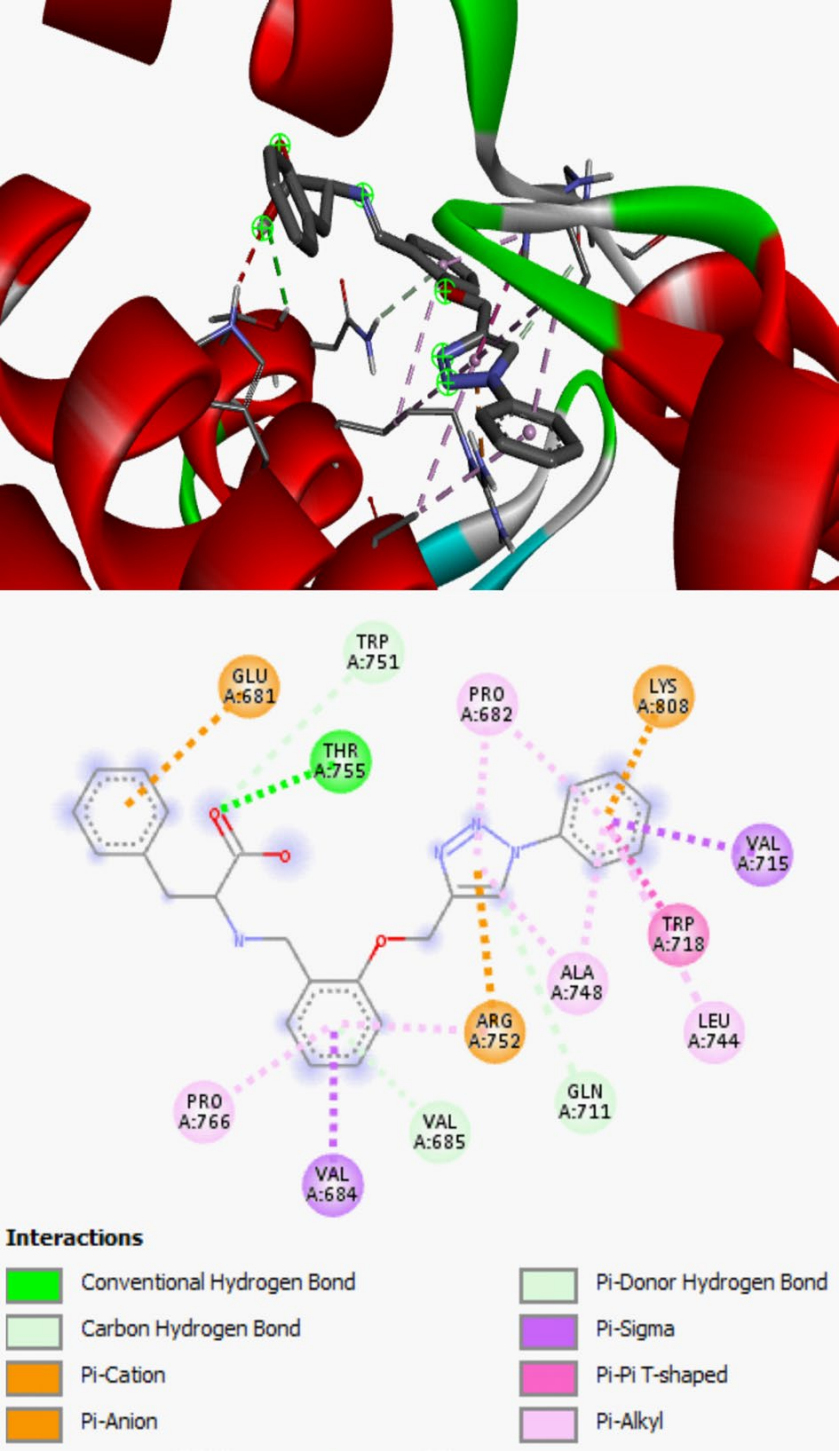
3D and 2D structure of compound **6** in the active site of Androgen receptor modulators.

The docking investigation on the studied compounds as Androgen receptor modulators (PDB ID: 5t8e) and Human MIA (PDB ID: 1i1j) inhibitors to down-regulate prostate and skin cancer were executed. The adequate choice of the docking target function impacts the accuracy of the ligand positioning as well as the accuracy of the protein–ligand binding energy calculation. In this work, the protein structure that is chemically similar to the studied compounds was selected for docking study and before the execution of the docking calculation, the flexibility of the selected protein was evaluated and the proteins with appropriate flexibility was selected for docking study. Also, the method of preparation and resolution (≤ 2 Å) of the studied target which agreed with the standard before subjected to further study were considered. The inhibiting activities of the studied synthesized compounds against the studied receptors were compared with the inhibiting activity of Cisplatin against Androgen receptor modulators and Human MIA. As shown in Table 5, the calculated binding affinities for the studied compounds were higher than the reported binding affinity for the referenced compound (Cisplatin). This showed that all the studied compounds proved to be potent in inhibiting Androgen receptor modulators and human MIA than Cisplatin. More so, compound **3** and **6** has proved to be more potent in inhibiting the targets than other studied compounds. This could be confirmed via the combination of amino acid residues and types of biological interactions involved in the docking study between compound **3** and **6** in the active site of Human MIA (PDB ID: 1i1j) complexes and Androgen receptor modulators (PDB ID: 5t8e), respectively. This docking result is in good agreement with the high anticancer activity for compounds **3** and **6** observed in the experimental *invitro* anticancer study. The type of interactions involved in compound **3—**Human MIA complexes and compound **6—**Androgen receptor modulators were observed to increase the level of stability and selectivity in the active site of the targets (Figs. 9 and 10).

**Table 5:** Calculated binding affinity and interaction involved between the studied complexes.

	Androgen receptor modulators (PDB ID: 5t8e)			Human MIA (PDB ID: 1i1j)		
	Residue involved in the interaction	Type of Interaction involved in the interaction	Binding Affinity (kcal/mol)	Residue involved in the interaction	Type of Interaction involved in the interaction	Binding Affinity (kcal/mol)
**1**	LEU805, TRP751, THR755, TYR763, VAL684, ARG752	Conventional Hydrogen Bond, Pi-Sigma, Pi-Pi T-Shaped, Pi-Alkyl	− 6.90	PHE49, CYS17, LEU76, ASP103, TYR78, SER50	Conventional Hydrogen Bond, Carbon Hydrogen Bond, Pi-Anion, Pi- Pi Stacked, Alkyl-Alkyl	− 6.50
**2**	ALA735, LYS905, LYS910, ASP819, TYR739, PRO817, LYS822	Conventional Hydrogen Bond, Pi-Cation, Pi-Anion, Pi-Alkyl	− 6.40	ARG75, ALA73, GLN44, VAL64, THR39, ALA32	Conventional Hydrogen Bond, Pi-Cation, Pi-Sigma, Pi-Alkyl	− 6.20
**3**	TRP751, GLU681, ASN756	Conventional Hydrogen Bond, Pi-Anion, Pi-Donor Hydrogen Bond	− 6.90	ARG75, CYS106, GLY77, LEU76, TYR78, TRP102	Conventional Hydrogen Bond, carbon hydrogen bond, Pi-Sigma, Pi-Pi Stacked,	− 8.70
**4**	ARG752, TYR763, TRP751, ASN756, THR755	Conventional Hydrogen Bond, Carbon Hydrogen Bond, Pi-Donor Hydrogen Bond, Pi-Sigma, Pi-Pi T-shaped,	− 7.10	SER50, GLY61, LYS10, ASP103, LEU76, PHE49, SER50	Conventional Hydrogen Bond, Carbon Hydrogen Bond, Pi-Cation, Pi-Sigma, Pi-Pi T-shaped, Pi-Anion	− 8.10
**5**	ARG752, LYS808, VAL685, TRP718, VAL684, VAL715,	Pi-Cation, Pi-Cation, Pi-Donor Hydrogen Bond, Pi-Sigma, Pi-Pi T-shaped	− 8.10	ASP103, LEU76, TRP102	Carbon Hydrogen Bond, Pi-Sigma, Pi-Pi T-shaped,	− 7.20
**6**	PRO682, ALA748, ARG752, GLY683, VAL684, ASN756, THR755, TRP751	Conventional Hydrogen Bond, carbon hydrogen bond, Unfavorable Donor-donor, Pi-Cation, Pi-Donor Hydrogen Bond, Amide-Pi Stacked, Pi-Alkyl	− 8.20	TYR78, ASP103, ARG36, LEU76, TRP103	Conventional Hydrogen Bond, carbon hydrogen bond, Pi-Sigma, Pi-Pi T-stacked, Pi-Pi T-shaped, Pi-Alky	− 8.10
Cisplatin	–	–	− 3.87	–	–	− 3.37

The calculated binding affinity for compound **1**–**6** were − 6.90 kcal/mol, − 6.40 kcal/mol, − 6.90 kcal/mol, − 7.10 kcal/mol, − 8.10 kcal/mol, − 8.20 kcal/mol for androgen receptor modulators (PDB ID: 5t8e) while − 6.50 kcal/mol, − 6.20 kcal/mol, − 8.70 kcal/mol, − 8.10 kcal/mol, − 7.20 kcal/mol, − 8.10 kcal/mol for human MIA.

### Absorption, distribution, metabolism, and excretion (ADME) prediction

Druglikeness evaluates whether a particular molecule is similar to the known drug or not. It is a complex balance of various properties and structural features of a compound. Lipinski’s rule is widely used to determine molecular properties that are important for drug’s pharmacokinetic in vivo. According to Lipinski’s rule of five, a candidate molecule is more likely to be orally active if: (a) MW ≤ 500, (b) MLogP ≤ 4.15, (c) HBD ≤ 5, (d) HBA ≤ 10, and (e) the number of violations ≤ 1^47^. These parameters were calculated by the online available swissADME web tool (http://www.swissadme.ch/) and are presented in Table 6. Low molecular weight drug molecules (< 500) are easily transported, diffuse, and absorbed as compared to heavy molecules. The molecular weight of all the compounds were found to be less than 500. Partition coefficient or Log P is an important parameter used in rational drug design to measure molecular hydrophobicity. Hydrophilic/lipophilic nature of drug molecule affects drug absorption, bioavailability, drug-receptor interactions, metabolism of molecules, as well as their toxicity. All the compounds exhibited MLog P value less than 4.15 which proved the lipophilic efficiency of the compounds. Lipophilicity plays an important role in the distribution of drug after absorption in the body. All the compounds have less than 5 and 10, hydrogen bond donors and acceptors, respectively which obeys the Lipinski’s rule of five. Topological polar surface area (TPSA) is closely related to the hydrogen bonding potential of a molecule and is a very good predictor of drug transport properties such as intestinal absorption, bioavailability, blood brain barrier penetration etc^48^. TPSA of all the compounds were found in the range of 87.83–126.90 and it is in the acceptable range of < 160 Å limit. Number of rotatable bonds is a simple topological parameter that measures molecular flexibility and is a good descriptor of oral bioavailability of drugs. The greater the number of rotatable bonds, the more flexible the molecule is to achieve different conformations. The number of rotatable bonds for the compounds were in the range of 9–11. The topological parameter and the number of rotatable bonds are considered to be good descriptors of the oral bioavailability of drugs^49^.

**Table 6:** In silico physicochemical data for drug likeness based on the Lipinski rule (SAR).

Compound	MR^a^	MLogP^b^	TPSA^c^	N_atoms_^d^	MW^e^	N_ON_^f^	N_OHNH_^g^	N_viol_^h^	N_rotb_^i^	Vol^j^
Rule	–	≤ 4.15	–	–	≤ 500	≤ 10	≤ 5	≤ 1	–	–
**1**	77.73	1.86	57.01	21	279.29	4	0	0	5	248.69
**2**	108.57	1.32	126.90	17	408.41	8	2	0	10	357.40
**3**	131.98	2.54	94.39	36	487.49	6	1	0	10	411.24
**4**	120.75	2.27	87.83	35	486.43	7	0	0	11	387.53
**5**	112.27	1.03	107.28	32	438.41	7	1	0	10	363.09
**6**	121.67	2.79	89.60	32	426.47	6	1	0	9	385.00

^a^Molar refractivity; ^b^octanol-water partition coefficient, calculated by methodology developed by Molinspiration; ^c^polar surface area; ^d^number of non-hydrogen atoms; ^e^molecular weight; ^f^number of hydrogen-bond acceptor (O and N atoms); ^g^number of hydrogen-bond donors (OH and NH atoms); ^h^number of “Rule of five” violations; ^i^number of rotable bonds; ^j^molecular volume.

The drug under study is supposed to bind with the biological target. The biological target can be any common protein such as ion channels, enzymes, and receptors. The biological target is also known as the drug target. The predicted bioactivity scores of screened compounds as well as their comparison with the standard drug for GPCR ligand, ion channel modulator, kinase inhibitor, nuclear receptor ligand, protease inhibitor and enzyme inhibitory activity was computed using Molinspiration cheminformatics software (freely available on https://molinspiration.com) and are summarized in Table S4. In general, if the bioactivity score (G protein-coupled receptor (GPCR) ligand, a kinase inhibitor, ion channel modulator, nuclear receptor ligand, protease inhibitor, and enzyme inhibitor) of the synthesized compounds is > − 0.5, then the drug is biologically active, but if the score is < − 0.5, then the drug is not active. The bioactivity scores, as provided in Table S4, showed that triazole **1** and the 1,2,3-triazole and chiral Schiff base hybrids **2**–**6** are active and confirmed their binding flexibilities^50^.

**Figure 11:**
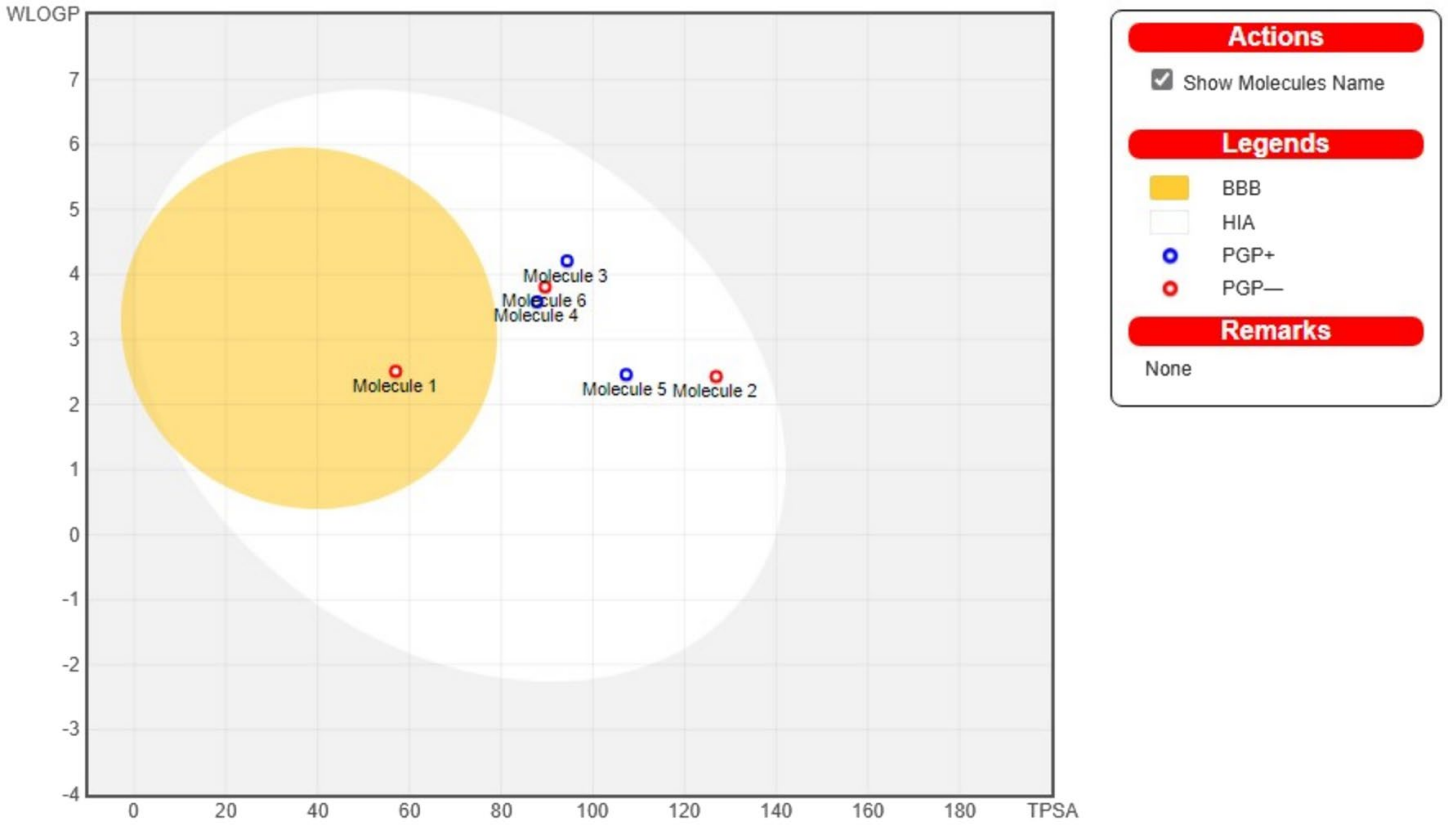
The BOILED-egg visualization predictive model for passive gastrointestinal absorption (hia) and blood–brain barrier (BBB) penetration of compounds **1**–**6** in the WLOGP-versus-TPSA diagram^51^.

The Swiss ADME software provides a ’BOILED-Egg’ visualization (Fig. 11) that displays two important ADME parameters: passive gastrointestinal absorption (HIA) and blood–brain barrier (BBB) access. These parameters mainly use two physicochemical descriptors: the octanol–water partition coefficient (MLogP) and the topological polar surface area (TPSA). In Fig. 11, the egg-shaped categorization plot depicts the white region, which represents the physiochemical space that favours HIA absorption, and the yolk region, which indicates properties that favour BBB permeability. Compounds **1** fall in the yolk region, indicating possible penetration of the BBB. In addition, it is likely that compounds with TPSA < 79 Å^2^ and relatively lipophilic properties reach the central nervous system (CNS). However, the ’BOILED-Egg’ approach is limited to passive molecules. The blue dots on the diagram indicate compounds that are likely to be removed from the CNS by p-glycoproteins, while the red 

dots indicate compounds that are likely to remain in the CNS. Compounds **2**–**6** exhibit favorable physicochemical properties with low molecular weights and TPSA values < 140 Å^2^, indicating good human intestinal absorption (HIA)^51^.

## Conclusion

The 1,2,3-triazole and chiral Schiff base hybrids **2**–**6** were successfully synthesized and their chemical structures were established using different spectroscopic techniques. Crystal of compound **1** was obtained and its structure was confirmed by single crystal X-ray diffraction. All compounds were evaluated for their anticancer activity against PC3 prostate cancer, skin cancer and MRC5 normal cells. All the compounds showed significant anticancer activity against the cancerous cells. Among the tested compounds **3** and **6** showed high activity for the inhibition of A375 and PC3 cancer cell lines and low toxicity for the healthy cell lines (MRC5). DFT study on the compounds proved the presence of electrophilic and nucleophilic bioactive sites for receptors. Molecular docking study proved that all the compounds are potent in inhibiting Androgen receptor modulators and human MIA than Cisplatin. The high binding affinity of compounds **3** and** 6** plays a vital role for their high anticancer activity observed in the experimental *invitro* anticancer evaluation. Structure activity relationships (SARs) of the tested compounds is in good agreement with DFT and molecular docking studies which proved that the presence of heterocyclic and lipophilic substituent on the Schiff base component of the hybrids is the main factor for high anticancer activity. The compounds exhibited desirable physicochemical properties for druglikeness. Thus, this preliminary study could be a foundation for researchers to gain more understanding on the synthesis and anticancer activity of 1,2,3-triazole and chiral Schiff base hybrids.

## Experimental

### Reagents

All the solvents used were analytical grade. All the reagents were purchased from Sigma Aldrich and were used as received without further purification unless otherwise stated. Salicylaldehyde (97%), potassium carbonate anhydrous (99.5%) (K_2_CO_3_), acetone (≥ 99.5%), *N, N’*-dimethylformamide (DMF, 99.8%), propargyl bromide (80 wt. % in toluene), sodium nitrite (NaNO_2_, ≥ 97%,), sodium azide (NaN_3_, ≥ 99.5%), copper(II) sulphate pentahydrate (CuSO4·5H_2_O, 99%), sodium *L*-ascorbate (≥ 98%), sulfuric acid (H_2_SO_4_, 98%), *L*-tyrosine (≥ 99%), Phenylalanine ethyl ester hydrochloride (≥ 99%), *L*-tryptophan (≥ 98%), *L*-histidine, *L*-glutamic acid, sodium hydroxide (NaOH, ≥ 98%), Chloroform-D (CDCl_3_, 99.8 Atom % D), dimethyl sulfoxide-d_6_ (DMSO-d_6_, 99.9 Atom % D).

### Instruments and methods

#### Analytical

Melting points were determined using a Reichert-Jung Thermovar hot-stage microscope and are uncorrected. Infrared spectra were recorded using Tensor 27 Bruker and Perkin Elmer FT-IR spectrum BX. High resolution mass spectra (HRMS) or mass spectra (MS) were carried out on a Waters Synapt G2 instrument at University of Pretoria, South Africa. All ^1^H NMR spectra were recorded on Bruker 500 MHz NMR spectrometer at ambient temperature and are reported as chemical shift δ in units of parts per million (ppm) with reference to the solvent (2.50 ppm for DMSO-d_6_ and 7.26 ppm for C(H)DCl_3_) or TMS (0.00 ppm). Multiplicities are presented as: s (singlet); d (doublet); t (triplet); dd (doublet of doublet); and m (multiplet). Coupling constants *J* values are expressed in Hz and the number of protons expressed as nH. ^13^C NMR spectra were obtained using Bruker 500 MHz NMR spectrometer at ambient temperature. Spectra are reported as chemical shift δ in units of parts per million (ppm) with reference to the deuterated solvent (39.81 ppm for DMSO-d_6_ or 77.16 ppm for CDCl_3_). The crystal was mounted on a glass fibre and used for the X–ray crystallographic analysis. The X-ray intensity data were collected on a Bruker Apex DUO 4 K CCD diffractometer area detector system, equipped with a graphite monochromator and Mo K_α_ fine-focus sealed tube (λ = 0.71073 Å) operated at 1.5 KW power (50 kV, 30 mA). The detector was placed at 4 cm from the crystal. Crystal temperature during the data collection was kept constant at 100 (2) K, using an Oxford 700 + series cryostream cooler.

### Synthesis

#### Synthesis of 2-((1-phenyl-1H-1,2,3-triazol-4-yl)methoxy)benzaldehyde (1)

To solution of 2-(prop-2-yn-1-yloxy)benzaldehyde (1.35 g, 8.44 mmol) in DMF: H_2_O (100 mL. 4:1) was added azidobenzene(1.21 mL, 10.13 mmol) followed by the addition of CuSO_4_.5H_2_O (0.0600 mmol, 15.0 mg dissolved in 200 µL of water) and sodium ascorbate (0.880 mmol, 174 mg dissolved in 800 µL of water). The reaction mixture was stirred under reflux for 24 h. The hot solution was poured into ice-water mixture, and the precipitate formed was filtered, washed with ice-water (3 × 25 mL). The solid product was dried in *vacuo* at 100 °C to provide compound **1** as colourless solid. The solid product collected was then recrystallised from ethanol (20 mL, by slow evaporation) and provided compound **5** as crystalline solid^44^. Yield (2.07 g, 7.43 mmol, 88%). m.p.: 147 –149 ºC. ^1^H NMR (500 MHz, CDCl_3_, δ): 10.49 (s, 1H, HC=O), 8.08 (s, 1H, H-triazole), 7.84 (d, 1H, *J* = 7.8 Hz, Ar), 7.73 (d, 2H, *J* = 7.5 Hz, Ar), 7.57 (m, 3H), 7.45 (t, 1H, *J* = 7.5 Hz, Ar), 7.21 (t, 1H, *J* = 8.5 Hz, Ar), 7.07 (t, 1H, *J* = 7.5 Hz, Ar), 5.42 (s, 2H, CH_2_OAr). ^13^C NMR (126 MHz, CDCl_3_, δ): 189.72 (C=O), 160.6 (Ar), 144.4 (N–*C*=CH), 137.1, 136.2, 130.0, 129.3, 125.5, 121.7, 121.3 (Ar), 120.9 (N–C=*C*H), 113.3 (Ar), 62.9 (*C*H_2_OAr). FTIR (ν in cm^−1^): 3150 (w), 3116 (w), 3076 (w), 2956 (w), 2922 (bs), 2796 (w), 2647 (w), 2447 (w), 1961 (w), 1869 (w), 1801 (w), 1675 (s), 1600 (s), 1504 (w), 1481 (m), 1458 (s), 1406 (m), 1338 (w), 1286 (s), 1241 (s), 1189 (m), 1161 (s), 1104 (w), 1046 (s), 989 (m), 903 (w), 846 (m), 823 (s), 754 (s), 686 (s). CHN analysis: Anal. Cald. for C_16_H_13_N_3_O_2_: C, 68.81%, H, 4.69%, N, 15.05%; Found: C, 68.04%, H, 4.82%, N, 14.71%. ESI-TOF-HRMS: [M + H]^+^ calculated for C_16_H_13_N_3_O_2_: 280.1087, found 280.1091.

### General procedure for the synthesis of 1,2,3-triazole and chiral Schiff bases hybrids (2–6)

The reaction was carried out following the literature reported procedure^52^. To a stirred solution of sodium hydroxide (0.043 g, 1.08 mmol) in 20 mL methanol at 80 °C was added two equivalents of the amines. When the combined reaction mixture completely dissolved, compound **1** (0.100 g, 0.358 mmol) was added and the colour of the reaction solution eventually changed to yellow. The reaction was stirred for 1 h at 80 °C. After the solution was cooled down to room temperature 150 mL aliquots of diethyl ether were added. A yellow precipitate appeared. The yellow precipitate was separated from the solution, washed several times with diethyl ether and dried *in vacuo*.

#### Synthesis of (s,e)-2-((2-((1-phenyl-1H-1,2,3-triazol-yl)methoxy)benzylidene)amino)pentanedioic acid (2)

White crystalline solid. Yield (0.121 g, 0.297 mmol, 83%). m.p.: 146–148 °C. ^1^H NMR (500 MHz, DMSO-d_6_, δ) 10.0 (s, 1H, COOH), 10.34 (s, 1H, COOH), 9.00 (s, 1H, HC=N), 7.92 (s, 2H, H-triazole and Ar), 7.70–7.12 (m, 8H, Ar), 5.43 (s, 2H, C*H*_2_OAr), 4.98 (s, 1H, N–C*H*–CH_2_), 3.15 (s, 4H, N–CH–C*H*_2_). ^13^C NMR (126 MHz, DMSO-d_6_, δ) 189.5 (C=O), 160.4 (C=N), 136.4, 130.0, 128.9, 128.0, 127.7, 122.9, 121.4 (Ar), 120.2 (N–C=*C*H), 114.3 (Ar), 62.3 (N–*C*H–CH_2_), 56.6 (*C*H_2_OAr), 48.6 (N–CH–*C*H_2_). IR assignments (ν in cm^−1^): 3479 (s), 3156 (m), 2922 (w), 2879 (w), 1664 (s), 1595 (s), 1507 (m), 1484 (s), 1466 (w), 1455 (s), 1404 (s), 1386 (s), 1335 (m), 1232 (s), 1186 (s), 1158 (s), 1103 (s), 1043 (s), 989 (m), 846 (s), 820 (s), 755 (s), 683 (s), 660 (m), 638 (m). CHN analysis: Anal. Cald. for C_21_H_20_N_4_O_5_: C, 61.76%, H, 4.94%, N, 13.72%; Found: C, 61.08%, H, 5.32%, N, 13.14%.

#### Synthesis of sodium (s,e)-3-(1H-indol-3-yl)-2-((2-((1-phenyl-1H-1,2,3-triazol-4-yl)methoxy)benzylidene)amino)propanoate (3)

Colourless solid. **Yield** (0.150 g,0.308 mmol, 86%). m.p.: 196–198 °C. ^1^H NMR: (500 MHz, DMSO-d_6_, δ) 10.68 (s, 1H, NH), 8.89 (s, 1H, HC=N), 8.31 (s, 1H, H-triazole), 7.90–7.86 (m, 3H, Ar), 7.57 (t, 2H, *J* = 7.20 Hz, Ar), 7.52 (d, 1H, *J* = 8.0 Hz, Ar), 7.46 (t, 1H, *J* = 7.0 Hz, Ar), 7.36 (d, 1H, *J* = 7.5 Hz, Ar), 7.22 (t, 2H, *J* = 8.0 Hz, Ar), 6.99–6.91 (m, 3H, Ar), 6.84 (t, 1H, *J* = 7.2 Hz, Ar), 5.21 (d, 2H, *J* = 8.0 Hz, C*H*_2_OAr), 3.85 (s, 1H, N–C*H*–CH_2_), 3.32 (s, 2H, *J* = 14.0 Hz, N–CH–C*H*_2_). ^13^C NMR (126 MHz, DMSO-d_6_, δ) 174.8 (C=O), 157.0 (C=N), 154.2 (Ar), 143.9 (N–*C*=CH), 136.6, 136.0, 131.3, 129.9, 128.8, 127.7, 127.2, 125.2, 123.1, 122.8, 121.0 (Ar), 120.3 (N–C=*C*H), 118.7, 117.2, 113.4, 113.0, 111.1 (Ar), 78.5 (N–*C*H–CH_2_), 61.9 (*C*H_2_OAr), 30.1 (N–CH–*C*H_2_). IR assignments (ν in cm^−1^): 3276 (bs), 2367 (w), 1595 (s), 1498 (m), 1435 (s), 1284 (s), 1238 (m), 1166 (m), 1100 (m), 1046 (m), 1000 (m), 878 (m), 832 (m), 755 (s), 689 (s). CHN analysis: Anal. Cald. for C_27_H_22_N5NaO_3_: C, 66.52%, H, 4.55%, N, 14.37%; Found: C, 66.13%, H, 4.96%, N, 13.98%.

#### Synthesis of sodium (s,e)-3-(4-oxidophenyl)-2-((2-((1-phenyl-1H-1,2,3-triazol-4-yl)methoxy)benzylidene)amino)propanoate (4)

Reddish brown coloured solid. Yield (0.135 g, 0.279 mmol, 78%). m.p.: 148–150 °C. ^1^H NMR (500 MHz, DMSO‑d_6_, δ) 8.07 (s, 1H, HC=N), 8.01 (s, 1H, H-triazole), 7.94–7.85 (m, 1H, Ar), 7.59 (t, 3H, *J* = 7.5 Hz, Ar), 7.50–7.44 (m, 2H, Ar), 7.36 (t, 1H, *J* = 7.8 Hz, Ar), 7.24 (d, 1H, *J* = 8.5 Hz, Ar), 6.98 (t, 1H, *J* = 7.5 Hz, Ar), 6.45 (d, 2H, *J* = 8.5 Hz, Ar), 5.95 (d, 2H, *J* = 8.0 Hz, Ar), 5.22 (s, 2H, C*H*_2_OAr), 3.56 (d, 1H, *J* = 6.5 Hz, N–C*H*–CH_2_), 2.88 (d, 1H, *J* = 11.0 Hz, N–CH–C*H*), 2.62 (s, 1H, N–CH–C*H*). ^13^C NMR (126 MHz, DMSO-d_6_, δ) 166.6, 162.4 (Ar), 157.3 (C=N), 144.3 (N–*C*=CH), 132.1, 129.9, 128.8, 128.1, 127.4, 126.8, 122.8 (Ar), 120.2 (N–C=*C*H), 113.6 (Ar), 69.8 (N–*C*H–CH_2_), 61.9 (*C*H_2_OAr), 35.8 (N–CH–*C*H_2_). IR assignments (ν in cm^−1^): 2359 (w), 1598 (s), 1487 (m), 1452 (m), 1375 (m), 1265 (s), 1106 (w), 1046 (m), 1003 (m), 829 (w), 755 (s), 689 (s). CHN analysis: Anal. Cald. for C_25_H_20_N_4_Na_2_O_4_: C, 61.73%, H, 4.14%, N, 11.52%; Found: C, 61.34%, H, 4.52%, N, 10.85%.

#### Synthesis of sodium (s,e)-3-(1 h-indol-3-yl)-2-((2-((1-phenyl-1H-1,2,3-triazol-4-yl)methoxy)benzylidene)amino)propanoate (5)

Brown coloured solid. Yield (0.117 g, 0.268 mmol, 75%). m.p.: 234–236 °C. ^1^H NMR (500 MHz, DMSO-d_6_, δ) 8.06 (s, 1H, HC=N), 8.01 (s, 1H, H-triazole), 7.58 (t, 3H, *J* = 8.0 Hz, Ar), 7.47–7.43 (m, 2H, Ar), 7.33 (d, 1H, *J* = 7.5 Hz, Ar), 7.10–7.02 (m, 3H, Ar), 6.88—6.66 (m, 2H, Ar), 5.40 (s, 2H, C*H*_2_OAr), 2.68 (s, 2H, N–C*H*–CH_2_), 2.63 (d, 2H, *J* = 11.0 Hz, N–CH–C*H*_2_). ^13^C NMR (126 MHz, DMSO-d_6_, δ) 192.1 (C=O), 137.0, 131.8, 130.4, 130.0, 129.1, 128.1, 125.7, 123.7, 100.9 (Ar). 59.7 (N–*C*H–CH_2_), 58.1 (*C*H_2_OAr), 55.5 (N–CH–*C*H_2_). IR assignments (ν in cm^−1^): 3245 (bs), 1584 (s), 1487 (w), 1449 (w), 1406 (s), 1341 (w), 1289 (w), 1224 (s), 1101 (w), 1046 (m), 1000 (w), 881 (m), 815 (m), 755 (s), 689 (s). CHN analysis: Anal. Cald. for C_22_H_19_N_6_NaO_3_: C, 60.27%, H, 4.37%, N, 19.17%; Found: C, 59.61%, H, 4.79%, N, 18.58%.

#### Synthesis of (e)-3-phenyl-2-((2-((1-phenyl-1H-1,2,3-triazol-4-yl)methoxy)benzylidene)amino)propanoic acid (6)

Phenylalanine ethyl ester hydrochloride (0.205 g, 0.358 mmol) was dissolved in ethanol and compound **1** (0.100 g, 0.358 mmol) was added to this solution. Sodium hydroxide solution (50% in water) was prepared and added to the solution to proceed the reaction in basic condition. The reaction mixture was refluxed at 50 °C for 7 h. The solution was cooled, and solvent was removed by rotary evaporator. The product was filtered, the precipitated product was washed with ethanol and dried. White crystalline solid. Yield (0.130 g, 0.304 mmol, 85%). m.p.: 230–232 °C. ^1^H NMR (500 MHz, DMSO-d_6_, δ) 10.42 (s, 1H, COOH), 9.02 (s, 1H, HC=N), 8.92 (s, 1H, H-triazole), 7.91 (t, 2H, *J* = 8.2 Hz, Ar), 7.72–7.67 (m, 2H, Ar), 7.60 (t, 2H, *J* = 7.2 Hz, Ar), 7.51–7.46 (m, 3H, Ar), 7.39 (d, 1H, *J* = 7.5 Hz, Ar), 7.34 (t, 1H, *J* = 7.0 Hz, Ar), 7.27 (d, 1H, *J* = 8.0 Hz, Ar), 7.12 (t, 1H, *J* = 7.2 Hz, Ar), 6.98 (t, 1H,* J* = 7.5 Hz, Ar), 5.55 (s, 1H, N–C*H*–CH_2_), 5.44 (s, 2H, C*H*_2_OAr), 5.26 (s, 2H, N–CH–C*H*_2_). ^13^C NMR (126 MHz, DMSO-d_6_, δ) 189.3 (C=O), 160.3 (C=N), 155.5 (Ar), 143.6 (N–*C*=CH), 136.5, 129.8, 127.6, 126.9, 124.6, 122.7, 121.2, 120.5 (Ar), 120.1 (N–C=*C*H), 114.2, 113.0 (Ar), 98.6 (N–*C*H–CH_2_), 62.2 (*C*H_2_OAr), 53.4 (N–CH–*C*H_2_). IR assignments (ν in cm^−1^): 3285 (bs), 3062 (w), 3027 (w), 2927 (w), 2358 (w), 1578 (s), 1489 (w), 1449 (w), 1409 (s), 1341 (m), 1238 (m), 1189 (w), 1158 (w), 1098 (w), 1078 (w), 1032 (m), 900 (m), 862 (m), 755 (s), 697 (s). CHN analysis: Anal. Cald. for C_25_H_22_N_4_O_3_: C, 70.41%, H, 5.20%, N, 13.14%; Found: C, 69.75%, H, 5.59%, N, 12.52%.

### Anticancer and cytotoxicity evaluation

#### Cell culture

PC3, A375, and MRC5 cell lines were purchased from ATCC and were cultured in Dulbecco’s Modified Eagle Media (DMEM) supplemented with 10% Fetal Bovine Serum (FBS) (Highveld biological), 1% gentamycin and 1% penicillin/streptomycin (Sigma, USA). Cells were maintained at 37 °C under 5% of carbon dioxide (CO_2_) and 95% relative humidity. The cells were trypsinized (0.1% trypsin) once reached 85% confluency and counted, plated in 96 well plates for treatment with various concentrations of the compounds **1**–**6**.

#### Assay background

The growth inhibitory effect and cytotoxicity of the compounds were tested in triplicate against two cancer cell lines (PC3 and A375) and one healthy cell line (MRC5), respectively using Alamar Blue assay.

#### AlamarBlue assay

PC3, A375, and MRC5 cell lines were cultured in 96 well tissue culture plates. A cell density of 2.5 × 104 cells in 90 µL of media per well was added to the plates and incubated overnight before treatment with a range of concentrations (5–100 µg/mL) of compounds **1**–**6**. The controls of the experiment were DMEM only (blank), cells treated with DMSO (0.1%), untreated cells in DMEM, and 100 µM cisplatin (Sigma Aldrich) (positive control). Cells were treated for 24 h. 10 µl of AlamarBlue (Thermo Fisher Scientific) was added to each well at 22 h after treatment and incubated for 2 h at 37 °C in the dark since AlamarBlue is light sensitive. Cell viability was measured using AlamarBlue and readings were obtained in terms of fluorescent values using a microplate reader (BioTek Synergy HT). Samples were exposed to an excitation wavelength of 530 nm and at the emission wavelength of 590 nm. Cell viability percentage was calculated using the formula below:

$$Cell \,viability=\frac{\mathrm{absorbance \,of \,treated \,cells}-\mathrm{absorbance \,of \,blank }}{\mathrm{absorbance \,of \,untreated \,cells }-\mathrm{ absorbance \,of \,blank}} \times 100\%$$

### Computational method

The Gauss View 5.0^53^ molecular builder and visualization software was used to construct input structures of Compounds 1–6. Geometry optimization of these structures was then executed at the B3LYP-gD3/6–311 +  + G(d,p) level of theory using Gaussian 16 Rev B.01 software^54^. The solvation environment was modelled with the default Polarizable Continuum Model (PCM) in Gaussian using methanol as solvent. Frequency calculations was carried out using the harmonic rotor approximation as implemented in Gaussian software. The absence of imaginary vibrational frequencies in the optimized structures confirmed them as true representative ground state structures of the compounds under investigation. Gauss View 5.0 software was subsequently used to generate and visualize the dipole moment vectors and output representations for the investigated compounds. Molecular Electrostatic Potential (MEP) map was generated using multiwfn software version 3.8^55,56^ while the Frontier Orbital energy plots and diagrams were obtained using Avogadro software^57^.

### Molecular docking study

The interactions between the studied compounds and Androgen receptor modulators (PDB ID: 5t8e)^58^ for prostate cancer as well as Human MIA (PDB ID: 1i1j)^59^ for skin cancer were investigated using molecular docking study. The study was executed using appropriate software such as Spartan 14 software^60^, Pymol v 1.7.4 software^61^, AutoDockTools-1.5.6^62^, Autodock vina software^63^ and discovery studio client v19.1.0.18287^64^. The studied compounds (**1**–**6**) were optimized using 6-31G* in order to obtain full geometry before subjecting to docking software. The optimized compounds were converted to .pdb format from. spartan format using Spartan 14 software which was further converted to .pdbqt using AutoDockTools-1.5.6. The studied receptors were retrieved from online protein database (https://www.rcsb.org/) and treated by removing small molecules as well as water molecules downloaded with the studied receptors using discovery studio client v19.1.0.18287. The docking of the studied compounds against the target requires locating the active site in the treated protein; therefore, the calculated value for the centre and size in X, Y and Z directions that show the located binding site were 20.163, 5.238, and 10.845 for the centre and 72, 62 and 74 for size (Androgen receptor modulators (PDB ID: 5t8e)) while 20.003, 21.173 and 1.583 for the center and 48, 50 and 40 for size described the active site of the Human MIA (PDB ID: 1i1j). The binding affinities for the studied complexes were accomplished using Autodock vina software before post-docking analysis.

### Accession codes

CCDC 2297996 contains the supplementary crystallographic data for this paper. These data can be obtained free of charge via www.ccdc.cam.ac.uk/data_request/cif, or by emailing data_request@ccdc.cam.ac.uk, or by contacting The Cambridge Crystallographic Data Centre, 12 Union Road, Cambridge CB2 1EZ, UK; fax: + 44 1223 336 033.

### Supplementary Information

Supplementary Information.

## Data Availability

All the findings are available in the manuscript and the supplementary material.
