# Supplementary material for: 1,2,3-triazole and chiral Schiff base hybrids as potential anticancer agents: DFT, molecular docking and ADME studies
Source: Sci Rep. 2024 Mar 23;14:6951. doi: 10.1038/s41598-024-57689-5 (PMC10960833; doi:10.1038/s41598-024-57689-5)
Supplement: Supplementary file 1 — Supplementary Information. [file 41598_2024_57689_MOESM1_ESM.docx]

**Supplementary Material**

**1,2,3-TRIAZOLE AND CHIRAL SCHIFF BASE HYBRIDS AS POTENTIAL ANTICANCER**

**AGENTS: DFT, MOLECULAR DOCKING AND ADME STUDIES**

***Yonas Belay ^a*^, Alfred Muller ^a^, Fanikie S. Mokoena ^a^, Adedapo S. Adeyinka ^a^, Lesetja R. Motadi ^b^, Abel K. Oyebamiji ^c^***

*^a^ Department of Chemical Sciences, University of Johannesburg, P.O. Box 524, Auckland Park, 2006, South*

*Africa. Email:* [*yhbelay@yahoo.com*](mailto:yhbelay@yahoo.com)

*^b^Department of Biochemistry, University of Johannesburg, P.O. Box 524, Auckland Park, 2006, South*

*Africa.*

*^c^Industrial Chemistry Programme, Bowen University, PMB 284, Iwo, Osun State, Nigeria.*


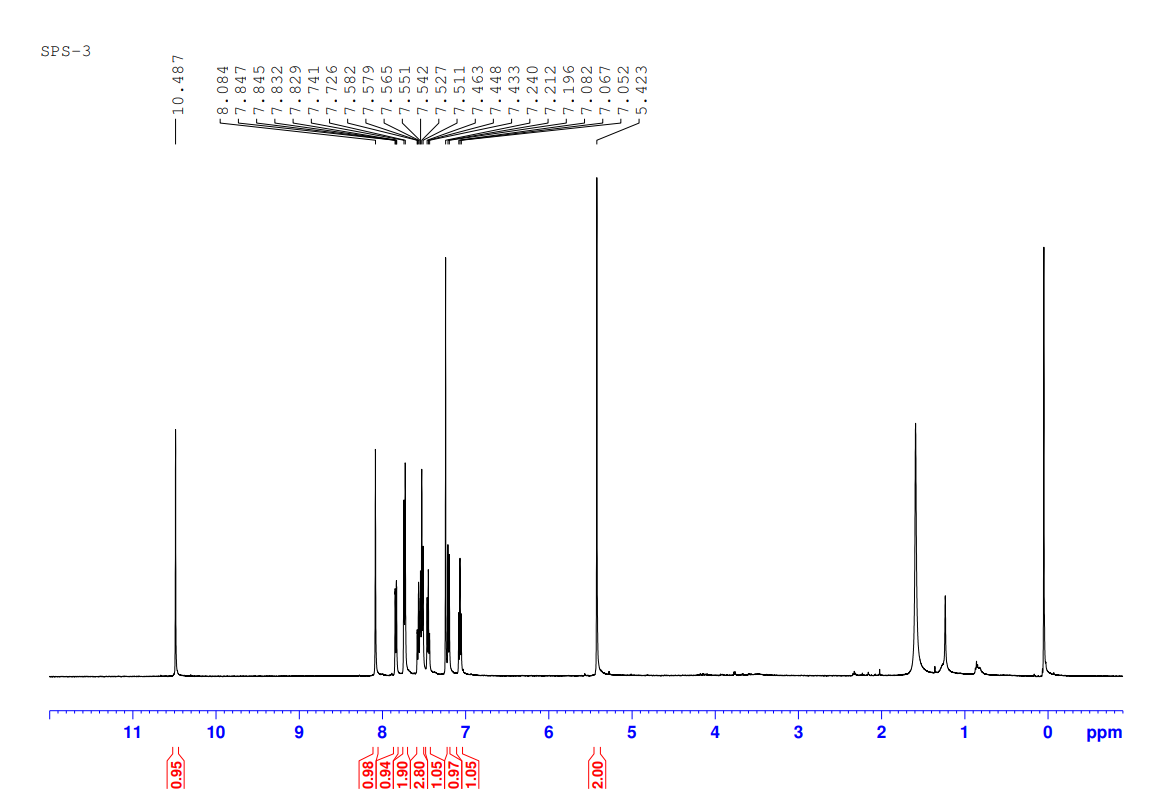


**H-triazole**

**Figure S1**. ^1^H NMR spectrum of compound **1**.


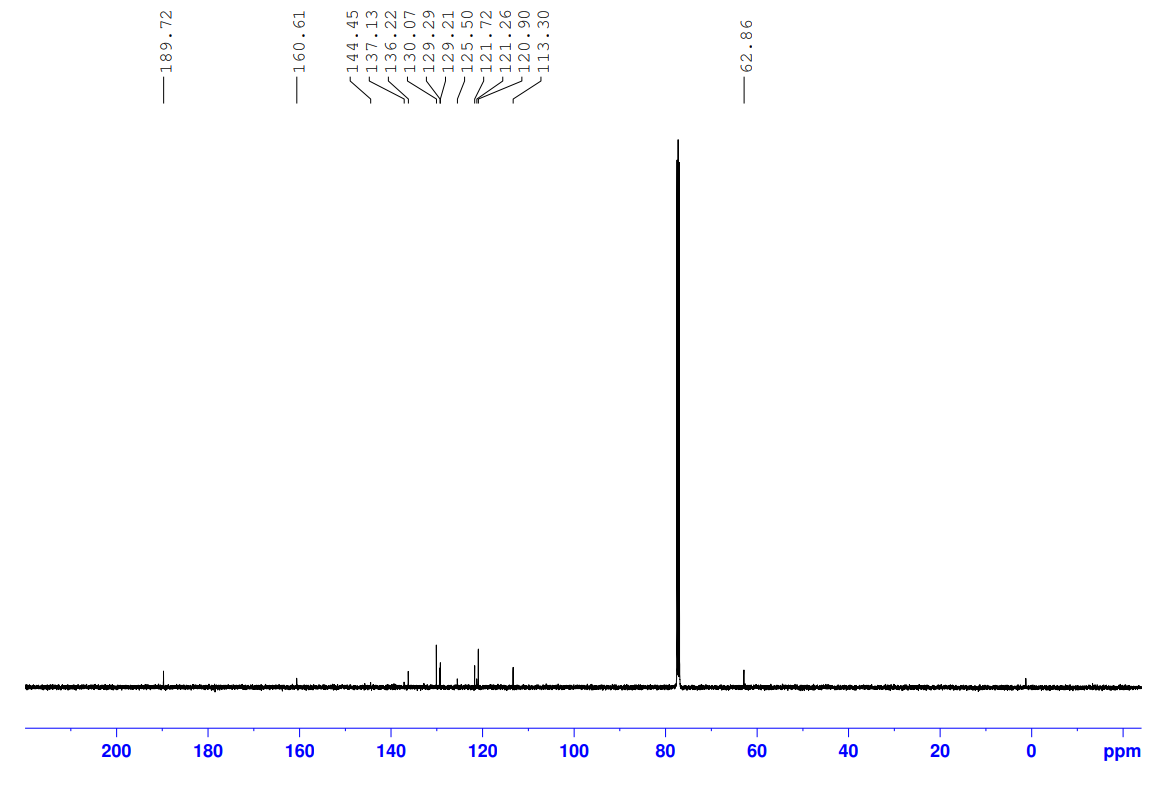


**Figure S2**. ^13^C NMR spectrum of compound **1**.

**Figure S3. ESI-TOF-HRMS:** [M+H]^+^ calculated for C_16_H_13­_N_3­_O_2_: 280.1087, found 280.1091.


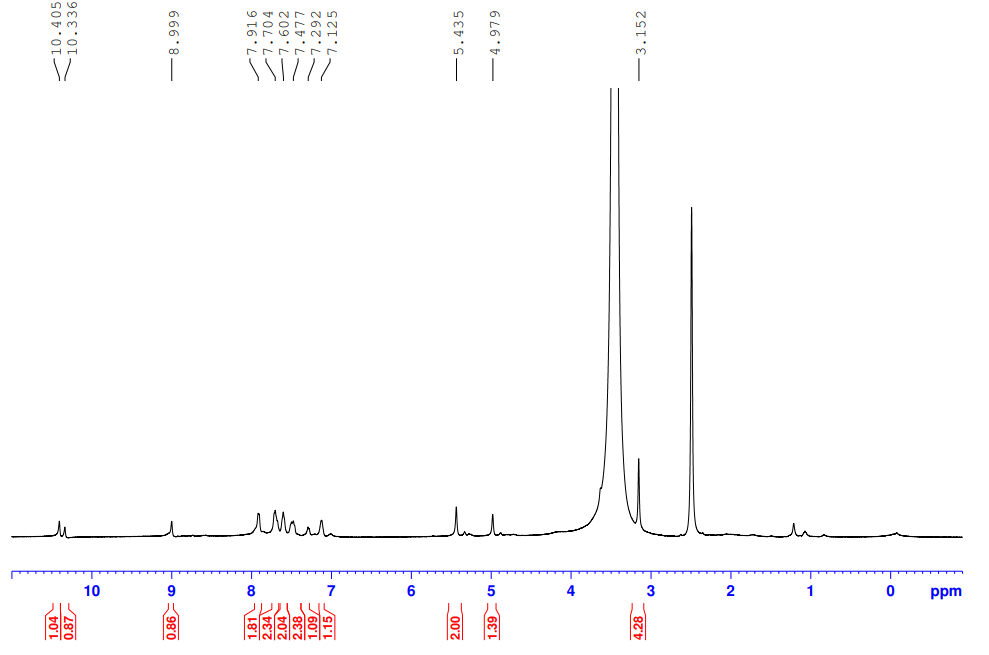


**DMSO-d_6_**

**OH**

**OH**

**HC=N**

**b**

**a**

**Figure S4**. ^1^H NMR spectrum of compound **2**.


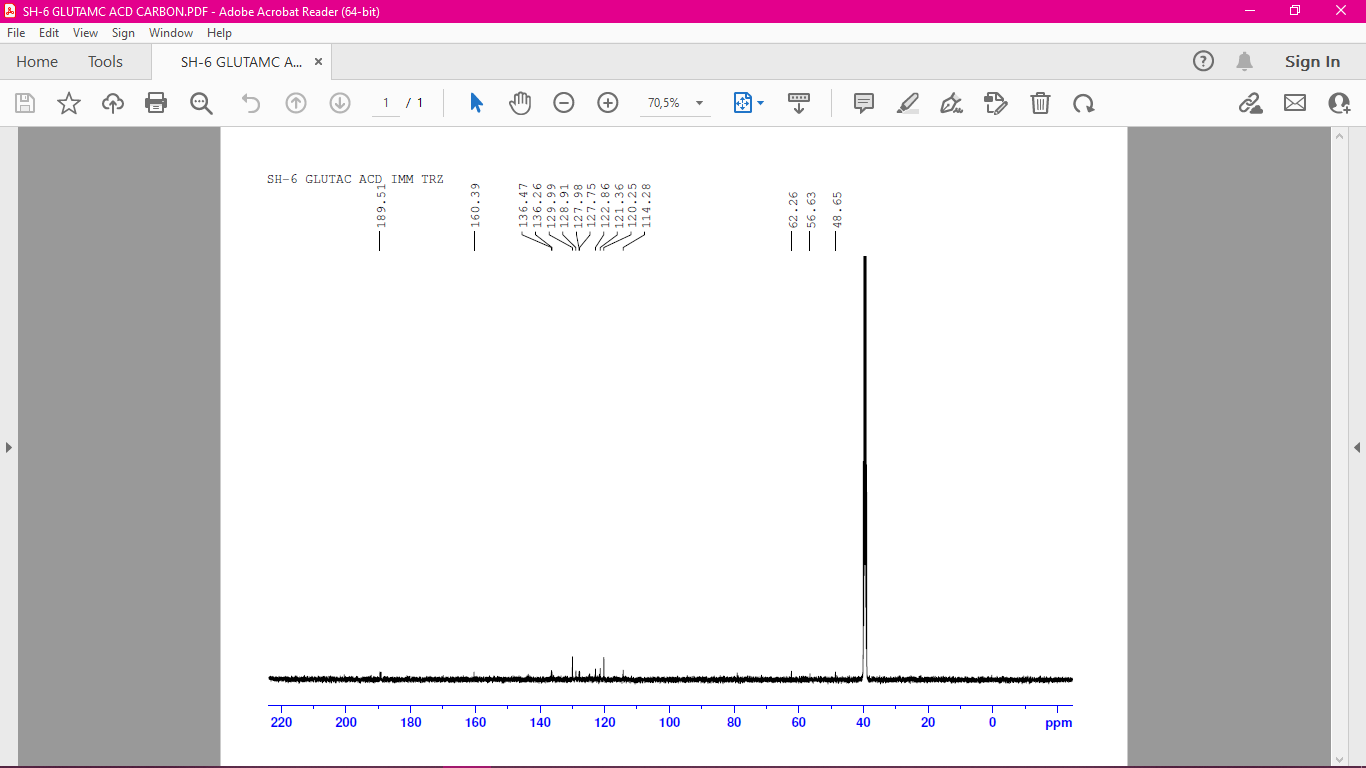

**Figure S5**. ^13^C NMR spectrum of compound **2**.


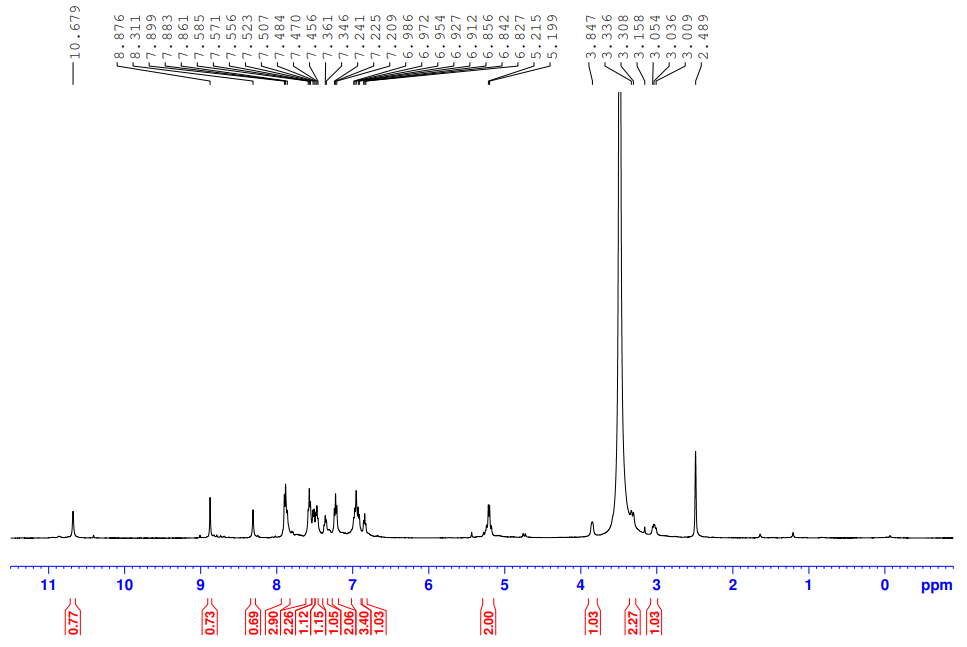


**DMSO-d_6_**

**b**

**HC=N**

**NH**

**a**

**Figure S6**. ^1^H NMR spectrum of compound **3**.


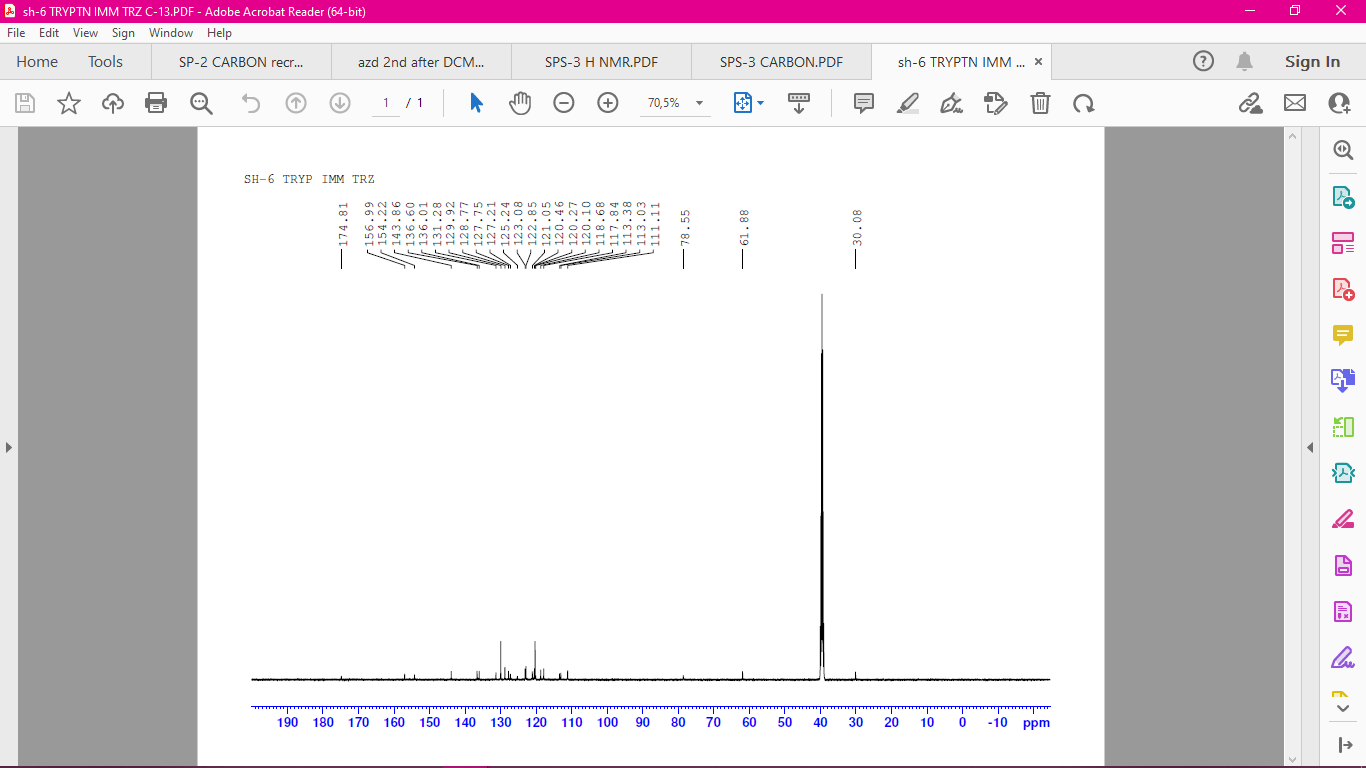

**Figure S7**. ^13^C NMR spectrum of compound **3**.


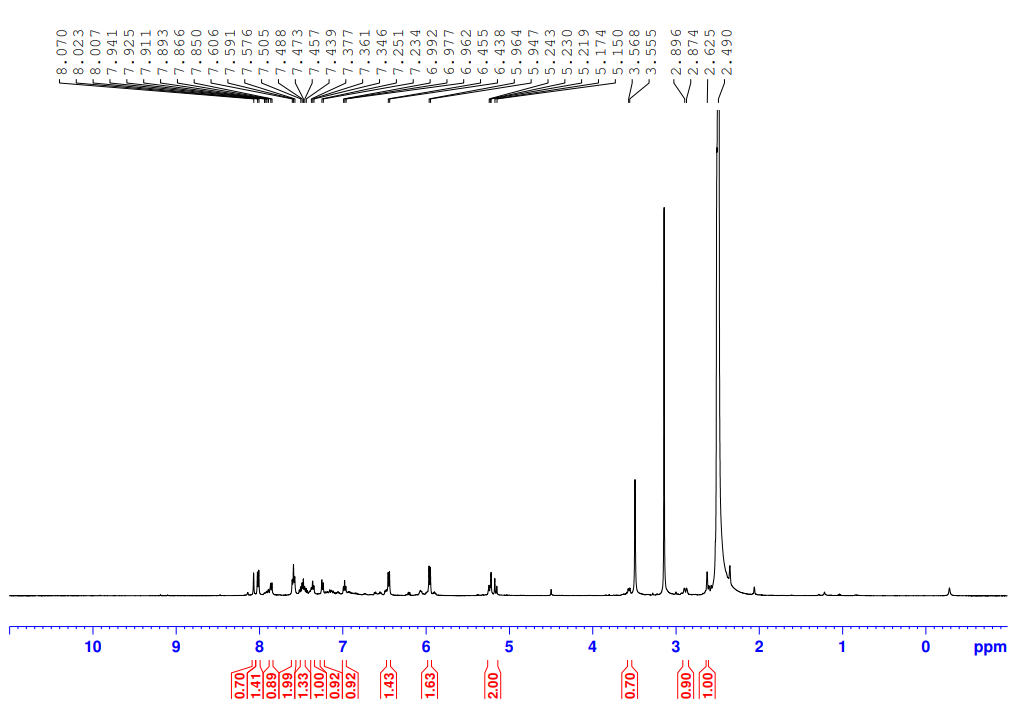


**DMSO-d_6_**

**b**

**a**

**c**

**HC=N**

**Figure S8**. ^1^H NMR spectrum of compound **4**.


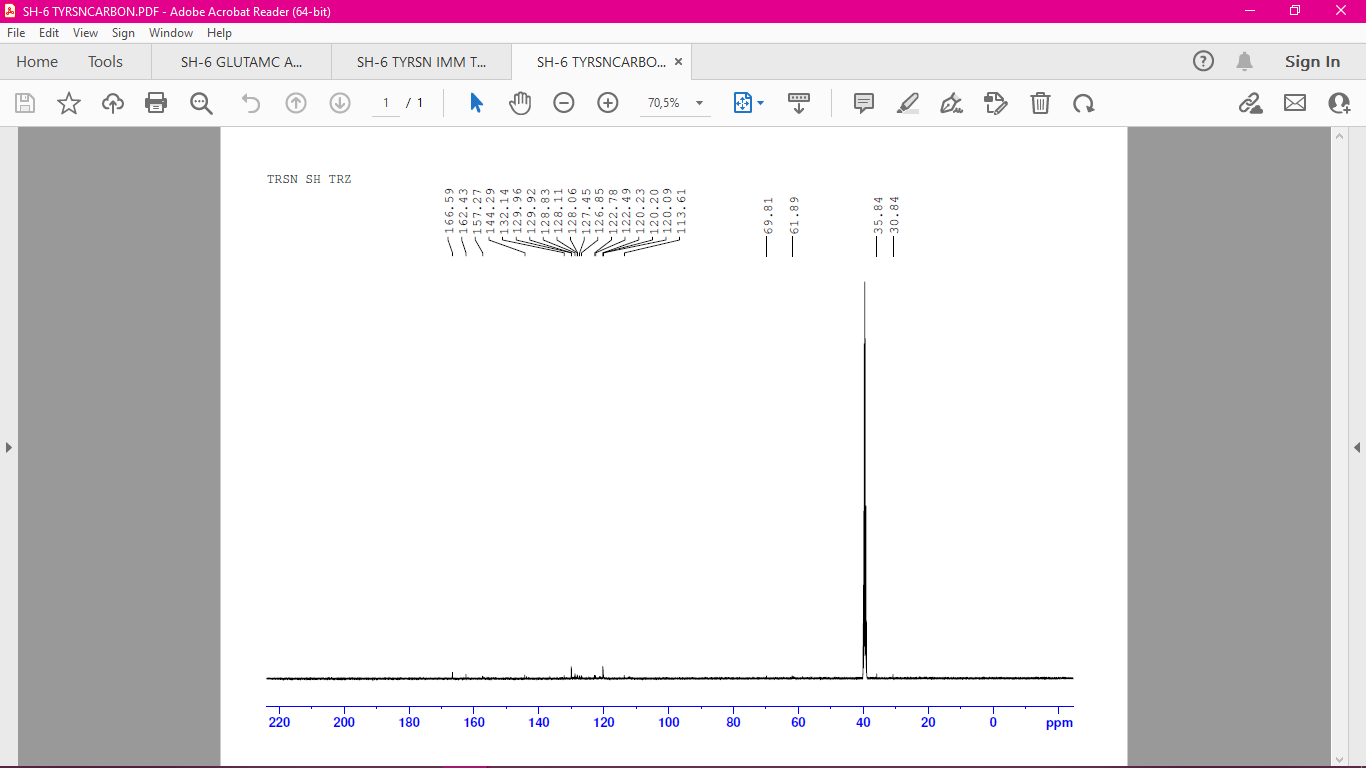

**Figure S9**. ^13^C NMR spectrum of compound **4**.

**
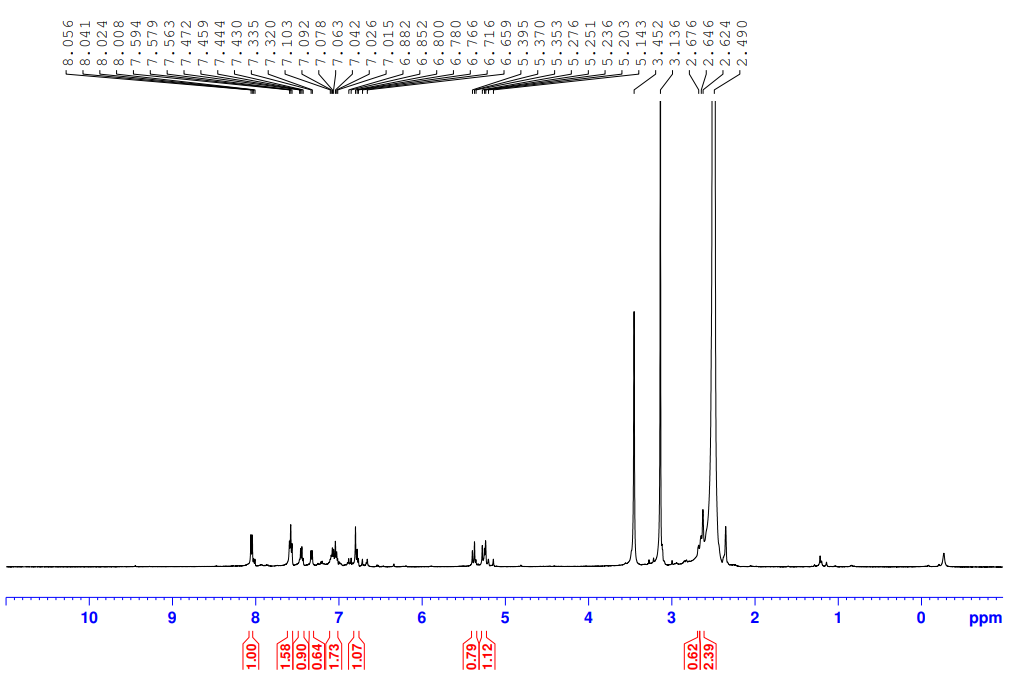
**

**DMSO-d_6_**

**a**

**b**

**HC=N**

**Figure S10**. ^1^H NMR spectrum of compound **5**.


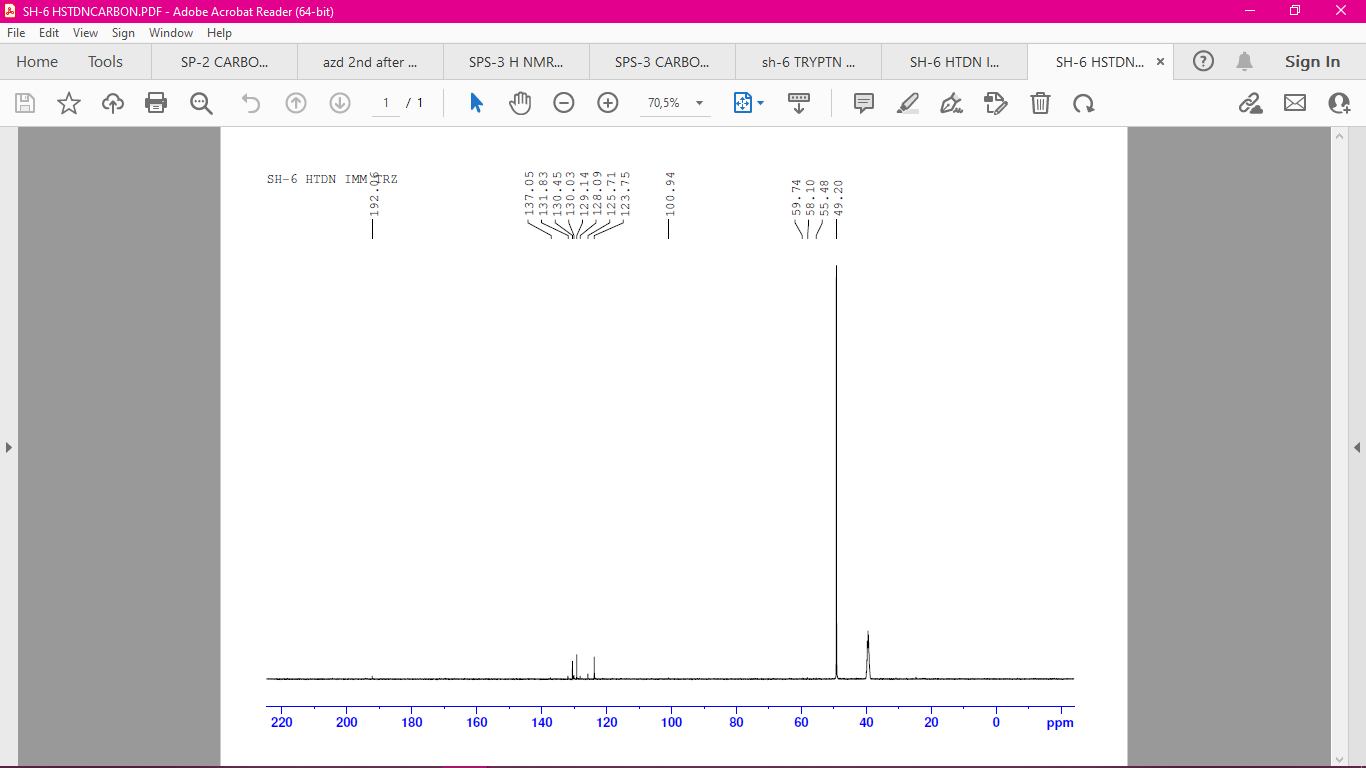

**Figure S11**. ^13^C NMR spectrum of compound **5**.


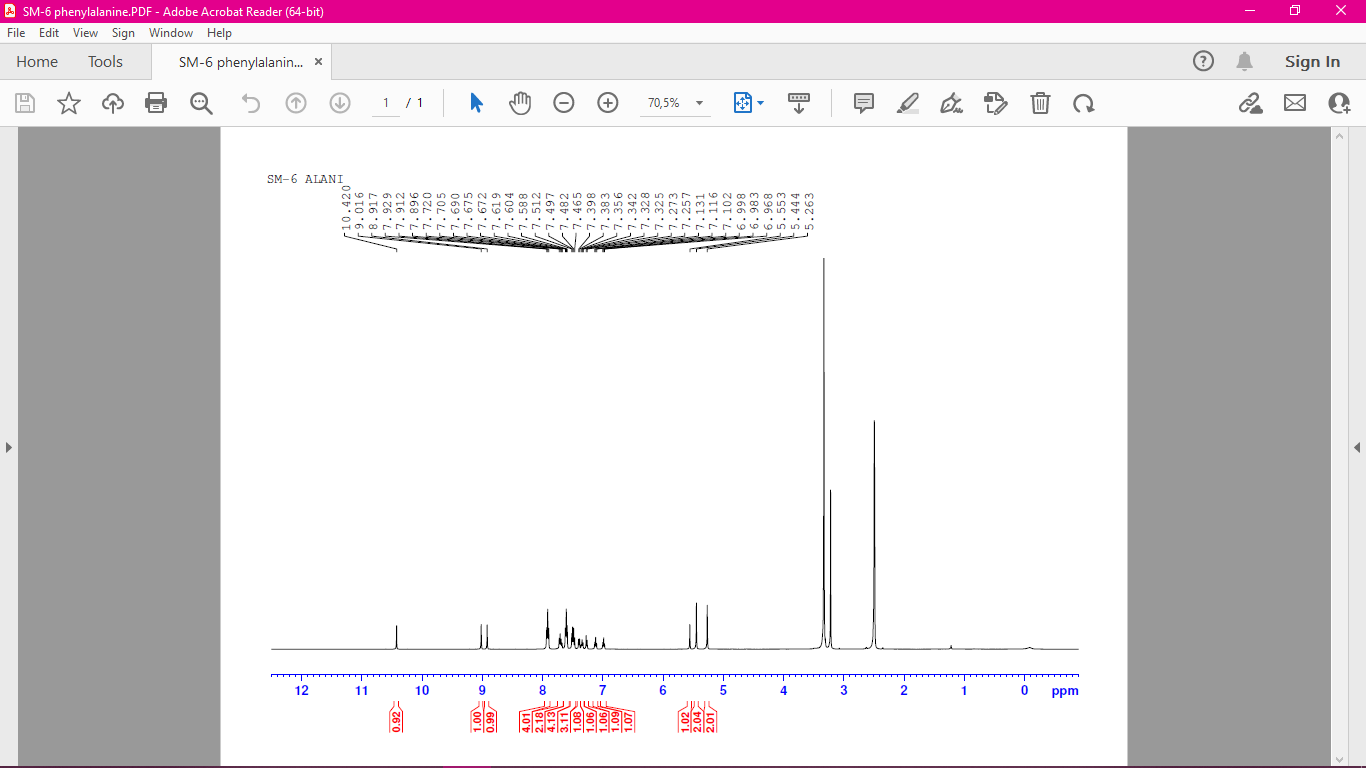


**DMSO-d_6_**

**b**

**a**

**HC=N**

**OH**

**Figure S12**. ^1^H NMR spectrum of compound **6**.


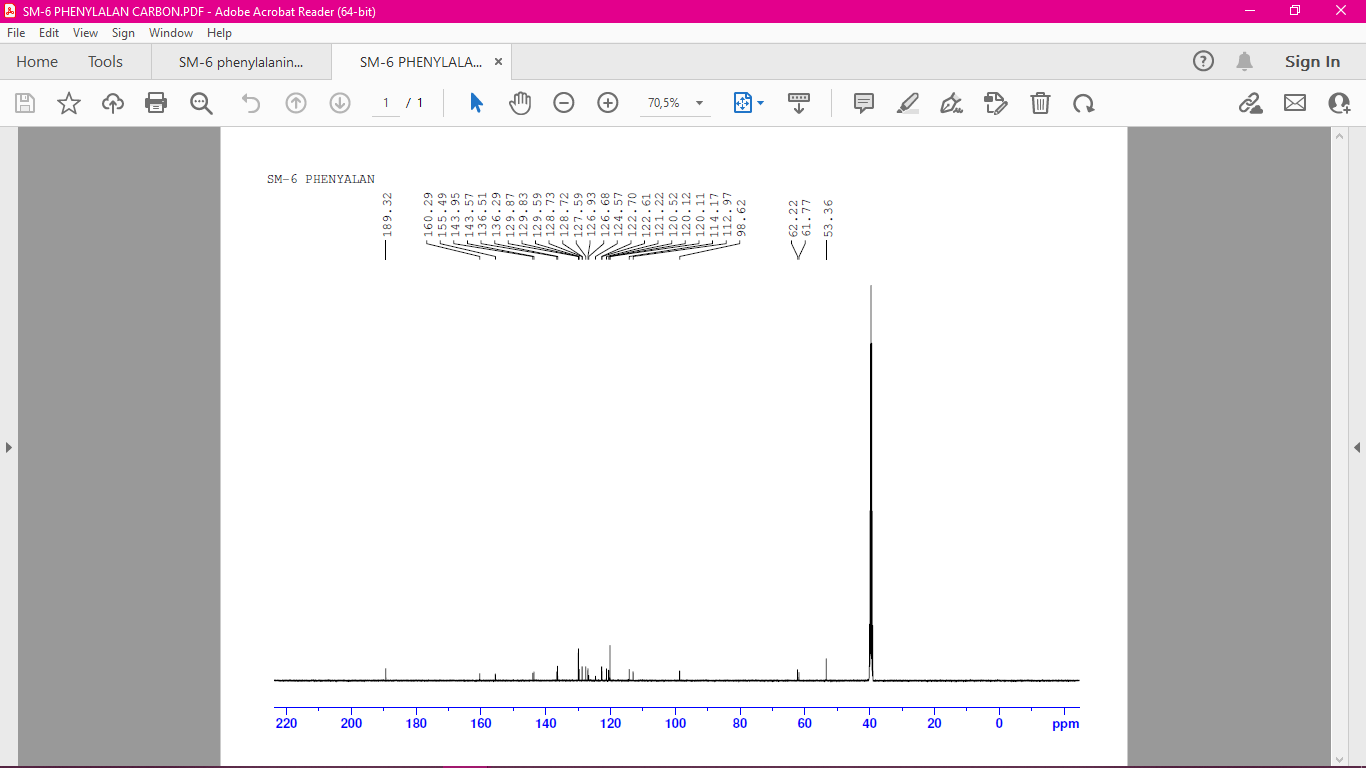

**Figure S13**. ^13^C NMR spectrum of compound **6**.

**Figure S14**. FTIR spectra of compounds **1** – **6**.

**Figure S15**. Graph showing cell viability of compounds **1** – **6** screened for cytotoxicity against PC3 cancer cell lines in the concentration range of 5 – 100 μg/mL.

**Figure S16**. Graph showing cell viability of compounds **1** – **6** screened for cytotoxicity against A375 cancer cell lines in the concentration range of 5 – 100 μg/mL.

**Table S1**. Hydrogen bonds for compound **1** [Å and °].

____________________________________________________________________________

D-H...A d(D-H) d(H...A) d(D...A) <(DHA)

____________________________________________________________________________

C(7)-H(12)...O(2)#1 0.95 2.39 3.311(5) 163.6

C(9)-H(10)...O(2)#1 0.95 2.63 3.451(5) 145.2

C(17)-H(14)...O(2)#1 0.95 2.48 3.320(5) 147.9

C(24)-H(20)...O(4)#2 0.95 2.38 3.299(5) 162.9

C(26)-H(21)...O(4)#2 0.95 2.63 3.447(5) 144.7

C(31)-H(18)...O(4)#2 0.95 2.47 3.321(5) 148.3

___________________________________________________________________________

**Table S2**. Cell viability and IC_50_ values of compounds **1** – **6** screened for cytotoxicity against PC3 cancer cell lines.

| **Compound** | **Cell viability (%)**  **Concentration (µg/mL)** | | | | | | **IC_50_ µg/mL** |
| --- | --- | --- | --- | --- | --- | --- | --- |
|  | **5** | **15** | **25** | **50** | **75** | **100** |  |
| **1** | 95 | 89 | 80 | 63 | 47 | 38 | 75.05 |
| **2** | \| 90.33 \| \| --- \| | 81 | 71 | 56 | 36 | 28 | 60.59 |
| **3** | 77 | 65.33 | 58 | 44 | 22 | 16 | 40.46 |
| **4** | 84 | 78 | 70 | 56 | 34 | 21.33 | 55.69 |
| **5** | 86 | 78.67 | \| 72.67 \| \| --- \| | 61.67 | 38 | 24 | 60.53 |
| **6** | 79 | 68 | 60 | 49 | 26 | 18 | 45.00 |
| **Cisplatin** | - | - | - | - | - | - | 30.11 |

**Table S3**. Cell viability and IC_50_ values of compounds **1** – **6** screened for cytotoxicity against A375 cancer cell lines.

| **Compound** | **Cell viability (%)**  **Concentration (µg/mL)** | | | | | | **IC_50_ µg/mL** |
| --- | --- | --- | --- | --- | --- | --- | --- |
|  | **5** | **15** | **25** | **50** | **75** | **100** |  |
| **1** | 79 | 69 | 55 | 37 | 24 | 18 | 40.37 |
| **2** | 75.67 | 67 | \| 53.33 \| \| --- \| | 34 | \| 20.67 \| \| --- \| | 14 | 36.12 |
| **3** | 68 | \| 57.67 \| \| --- \| | 42 | 23 | 14 | 9 | 21.86 |
| **4** | \| 68.67 \| \| --- \| | \| 59.67 \| \| --- \| | 45 | 25 | 16 | 11 | 24.83 |
| **5** | 72 | 61.33 | 47.33 | 29 | 18 | 10 | 28.94 |
| **6** | 68 | \| 57.67 \| \| --- \| | 45 | 27 | 16 | 8 | 24.18 |
| **Cisplatin** | - | - | - | - | - | - | 30.11 |

**Table S4**. Molinspiration bioactivity score of the synthesized compounds **1** - **6**.

| **Compound** | **GPCR^a^** | **ICM^b^** | **KI^c^** | **NRL^d^** | **PI^e^** | **EI^f^** |
| --- | --- | --- | --- | --- | --- | --- |
| **1** | -0.04 | -0.06 | -0.08 | -0.18 | -0.38 | 0.06 |
| **2** | 0.04 | -0.04 | -0.25 | -0.20 | -0.12 | 0.14 |
| **3** | 0.11 | 0.00 | 0.01 | -0.26 | -0.14 | 0.10 |
| 4 | -0.02 | -0.04 | -0.13 | -0.27 | -0.17 | 0.05 |
| **5** | 0.20 | 0.09 | 0.03 | -0.71 | -0.01 | 0.29 |
| **6** | 0.08 | -0.02 | -0.22 | -0.14 | -0.10 | 0.16 |

^a^G-protein coupled receptor; ^b^ion channel modulator; ^c^kinase inhibitor; ^d^nuclear receptor ligand; ^e^protease inhibitor; ^f^enzyme inhibitor.

**Absorption, Distribution, Metabolism, and Excretion**

**(ADME) Prediction**

**Compound 1**


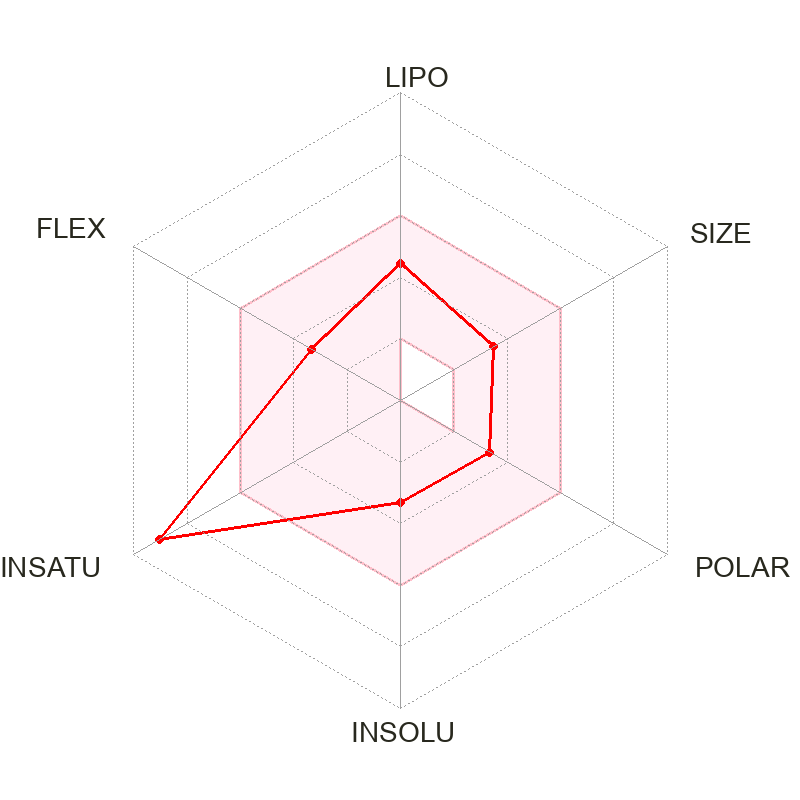


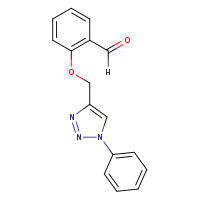


| SMILES | O=Cc1ccccc1OCc1nnn(c1)c1ccccc1 |
| --- | --- |

| **Physicochemical Properties** | |
| --- | --- |
| Formula | C_16_H_13_N_3_O_2_ |
| Molecular weight | 279.29 g/mol |
| Num. heavy atoms | 21 |
| Num. arom. heavy atoms | 17 |
| Fraction Csp3 | 0.06 |
| Num. rotatable bonds | 5 |
| Num. H-bond acceptors | 4 |
| Num. H-bond donors | 0 |
| Molar Refractivity | 77.73 |
| TPSA [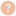](http://www.swissadme.ch/index.php) | 57.01 Å² |
| **Lipophilicity** | |
| Log *P*_o/w_ (iLOGP) [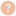](http://www.swissadme.ch/index.php) | 2.59 |
| Log *P*_o/w_ (XLOGP3) [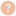](http://www.swissadme.ch/index.php) | 2.31 |
| Log *P*_o/w_ (WLOGP) [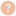](http://www.swissadme.ch/index.php) | 2.51 |
| Log *P*_o/w_ (MLOGP) [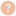](http://www.swissadme.ch/index.php) | 1.86 |
| Log *P*_o/w_ (SILICOS-IT) [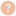](http://www.swissadme.ch/index.php) | 2.54 |
| Consensus Log *P*_o/w_ [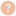](http://www.swissadme.ch/index.php) | 2.36 |
| **Water Solubility** | |
| Log *S* (ESOL) [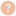](http://www.swissadme.ch/index.php) | -3.30 |
| Solubility | 1.41e-01 mg/ml ; 5.06e-04 mol/l |
| Class [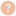](http://www.swissadme.ch/index.php) | Soluble |
| Log *S* (Ali) [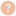](http://www.swissadme.ch/index.php) | -3.15 |
| Solubility | 2.00e-01 mg/ml ; 7.15e-04 mol/l |
| Class [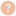](http://www.swissadme.ch/index.php) | Soluble |
| Log *S* (SILICOS-IT) [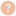](http://www.swissadme.ch/index.php) | -5.16 |
| Solubility | 1.92e-03 mg/ml ; 6.87e-06 mol/l |
| Class [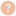](http://www.swissadme.ch/index.php) | Moderately soluble |
| **Pharmacokinetics** | |
| GI absorption [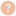](http://www.swissadme.ch/index.php) | High |
| BBB permeant [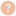](http://www.swissadme.ch/index.php) | Yes |
| P-gp substrate [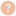](http://www.swissadme.ch/index.php) | No |
| CYP1A2 inhibitor [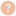](http://www.swissadme.ch/index.php) | Yes |
| CYP2C19 inhibitor [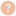](http://www.swissadme.ch/index.php) | Yes |
| CYP2C9 inhibitor [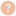](http://www.swissadme.ch/index.php) | Yes |
| CYP2D6 inhibitor [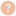](http://www.swissadme.ch/index.php) | No |
| CYP3A4 inhibitor [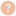](http://www.swissadme.ch/index.php) | No |
| Log *K*_p_ (skin permeation) [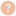](http://www.swissadme.ch/index.php) | -6.36 cm/s |
| **Druglikeness** | |
| Lipinski [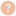](http://www.swissadme.ch/index.php) | Yes; 0 violation |
| Ghose [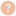](http://www.swissadme.ch/index.php) | Yes |
| Veber [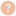](http://www.swissadme.ch/index.php) | Yes |
| Egan [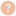](http://www.swissadme.ch/index.php) | Yes |
| Muegge [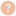](http://www.swissadme.ch/index.php) | Yes |
| Bioavailability Score [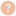](http://www.swissadme.ch/index.php) | 0.55 |
| **Medicinal Chemistry** | |
|  |  |
| PAINS [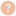](http://www.swissadme.ch/index.php) | 0 alert |
| Brenk [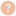](http://www.swissadme.ch/index.php) | 1 alert: aldehyde [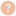](http://www.swissadme.ch/index.php) |
| Leadlikeness [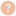](http://www.swissadme.ch/index.php) | Yes |
| Synthetic accessibility [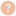](http://www.swissadme.ch/index.php) | 2.52 |

**Compound 2**

Bottom of Form


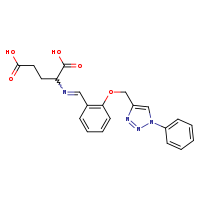


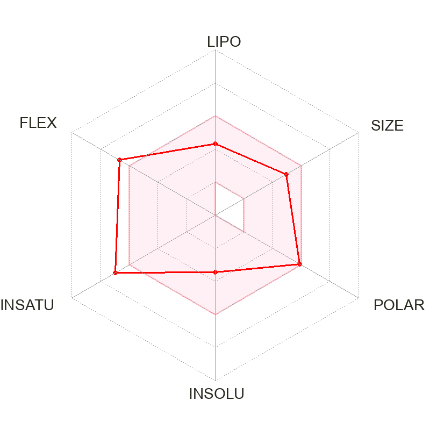


Top of Form

| SMILES | OC(=O)CCC(C(=O)O)/N=C/c1ccccc1OCc1nnn(c1)c1ccccc1 |
| --- | --- |

| Physicochemical Properties | |
| --- | --- |
| Formula | C_21_H_20_N_4_O_5_ |
| Molecular weight | 408.41 g/mol |
| Num. heavy atoms | 30 |
| Num. arom. heavy atoms | 17 |
| Fraction Csp3 | 0.19 |
| Num. rotatable bonds | 10 |
| Num. H-bond acceptors | 8 |
| Num. H-bond donors | 2 |
| Molar Refractivity | 108.57 |
| TPSA [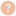](http://www.swissadme.ch/index.php) | 126.90 Å² |
| Lipophilicity | |
| Log *P*_o/w_ (iLOGP) [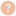](http://www.swissadme.ch/index.php) | 2.35 |
| Log *P*_o/w_ (XLOGP3) [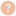](http://www.swissadme.ch/index.php) | 2.07 |
| Log *P*_o/w_ (WLOGP) [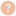](http://www.swissadme.ch/index.php) | 2.43 |
| Log *P*_o/w_ (MLOGP) [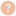](http://www.swissadme.ch/index.php) | 1.32 |
| Log *P*_o/w_ (SILICOS-IT) [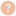](http://www.swissadme.ch/index.php) | 2.47 |
| Consensus Log *P*_o/w_ [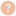](http://www.swissadme.ch/index.php) | 2.13 |
| Water Solubility | |
| Log *S* (ESOL) [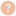](http://www.swissadme.ch/index.php) | -3.44 |
| Solubility | 1.50e-01 mg/ml ; 3.67e-04 mol/l |
| Class [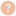](http://www.swissadme.ch/index.php) | Soluble |
| Log *S* (Ali) [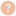](http://www.swissadme.ch/index.php) | -4.36 |
| Solubility | 1.77e-02 mg/ml ; 4.32e-05 mol/l |
| Class [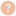](http://www.swissadme.ch/index.php) | Moderately soluble |
| Log *S* (SILICOS-IT) [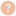](http://www.swissadme.ch/index.php) | -4.83 |
| Solubility | 6.04e-03 mg/ml ; 1.48e-05 mol/l |
| Class [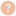](http://www.swissadme.ch/index.php) | Moderately soluble |
| Pharmacokinetics | |
| GI absorption [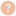](http://www.swissadme.ch/index.php) | High |
| BBB permeant [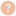](http://www.swissadme.ch/index.php) | No |
| P-gp substrate [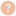](http://www.swissadme.ch/index.php) | No |
| CYP1A2 inhibitor [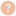](http://www.swissadme.ch/index.php) | No |
| CYP2C19 inhibitor [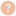](http://www.swissadme.ch/index.php) | Yes |
| CYP2C9 inhibitor [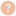](http://www.swissadme.ch/index.php) | Yes |
| CYP2D6 inhibitor [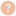](http://www.swissadme.ch/index.php) | No |
| CYP3A4 inhibitor [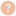](http://www.swissadme.ch/index.php) | No |
| Log *K*_p_ (skin permeation) [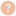](http://www.swissadme.ch/index.php) | -7.32 cm/s |
| Druglikeness | |
| Lipinski [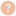](http://www.swissadme.ch/index.php) | Yes; 0 violation |
| Ghose [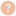](http://www.swissadme.ch/index.php) | Yes |
| Veber [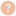](http://www.swissadme.ch/index.php) | Yes |
| Egan [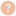](http://www.swissadme.ch/index.php) | Yes |
| Muegge [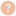](http://www.swissadme.ch/index.php) | Yes |
| Bioavailability Score [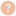](http://www.swissadme.ch/index.php) | 0.56 |
| Medicinal Chemistry | |
|  |  |
| PAINS [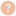](http://www.swissadme.ch/index.php) | 0 alert |
| Brenk [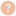](http://www.swissadme.ch/index.php) | 1 alert: imine_1 [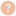](http://www.swissadme.ch/index.php) |
| Leadlikeness [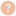](http://www.swissadme.ch/index.php) | No; 2 violations: MW>350, Rotors>7 |
| Synthetic accessibility [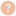](http://www.swissadme.ch/index.php) | 3.86 |

**Compound 3**


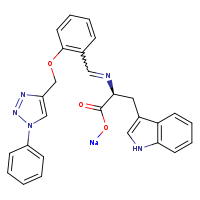

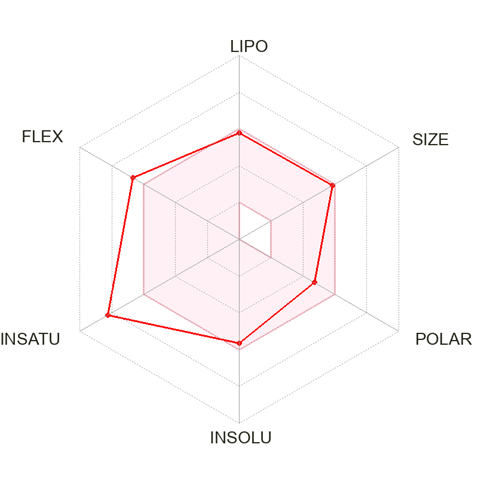


| SMILES | [Na]OC(=O)[C@H](Cc1c[nH]c2c1cccc2)/N=C/c1ccccc1OCc1nnn(c1)c1ccccc1 |
| --- | --- |

| Physicochemical Properties | |
| --- | --- |
| Formula | C_27_H_22_N_5_NaO_3_ |
| Molecular weight | 487.49 g/mol |
| Num. heavy atoms | 36 |
| Num. arom. heavy atoms | 26 |
| Fraction Csp3 | 0.11 |
| Num. rotatable bonds | 10 |
| Num. H-bond acceptors | 6 |
| Num. H-bond donors | 1 |
| Molar Refractivity | 131.98 |
| TPSA [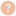](http://www.swissadme.ch/index.php) | 94.39 Å² |
| Lipophilicity | |
| Log *P*_o/w_ (iLOGP) [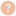](http://www.swissadme.ch/index.php) | 0.00 |
| Log *P*_o/w_ (XLOGP3) [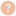](http://www.swissadme.ch/index.php) | 4.61 |
| Log *P*_o/w_ (WLOGP) [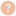](http://www.swissadme.ch/index.php) | 4.21 |
| Log *P*_o/w_ (MLOGP) [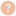](http://www.swissadme.ch/index.php) | 2.54 |
| Log *P*_o/w_ (SILICOS-IT) [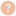](http://www.swissadme.ch/index.php) | 3.61 |
| Consensus Log *P*_o/w_ [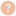](http://www.swissadme.ch/index.php) | 2.99 |
| Water Solubility | |
| Log *S* (ESOL) [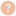](http://www.swissadme.ch/index.php) | -5.64 |
| Solubility | 1.11e-03 mg/ml ; 2.28e-06 mol/l |
| Class [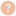](http://www.swissadme.ch/index.php) | Moderately soluble |
| Log *S* (Ali) [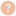](http://www.swissadme.ch/index.php) | -6.32 |
| Solubility | 2.35e-04 mg/ml ; 4.82e-07 mol/l |
| Class [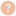](http://www.swissadme.ch/index.php) | Poorly soluble |
| Log *S* (SILICOS-IT) [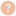](http://www.swissadme.ch/index.php) | -8.77 |
| Solubility | 8.34e-07 mg/ml ; 1.71e-09 mol/l |
| Class [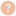](http://www.swissadme.ch/index.php) | Poorly soluble |
| Pharmacokinetics | |
| GI absorption [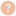](http://www.swissadme.ch/index.php) | High |
| BBB permeant [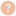](http://www.swissadme.ch/index.php) | No |
| P-gp substrate [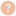](http://www.swissadme.ch/index.php) | Yes |
| CYP1A2 inhibitor | No |
| CYP2C19 inhibitor | Yes |
| CYP2C9 inhibitor | Yes |
| CYP2D6 inhibitor | Yes |
| CYP3A4 inhibitor | Yes |
| Log *K*_p_ (skin permeation) | -6.00 cm/s |
| Druglikeness | |
| Lipinski | Yes; 0 violation |
| Ghose | No; 2 violations: MW>480, MR>130 |
| Veber | Yes |
| Egan | Yes |
| Muegge | Yes |
| Bioavailability Score | 0.55 |
| Medicinal Chemistry | |
|  |  |
| PAINS | 0 alert |
| Brenk | 1 alert: imine_1 |
| Leadlikeness | No; 3 violations: MW>350, Rotors>7, XLOGP3>3.5 |
| Synthetic accessibility | 4.25 |

**Compound 4**

| SMILES | [Na]OC(=O)[C@H](Cc1ccc(cc1)O[Na])/N=C/c1ccccc1OCc1nnn(c1)c1ccccc1 |
| --- | --- |

| Physicochemical Properties | |
| --- | --- |
| Formula | C_25_H_20_N_4_Na_2_O_4_ |
| Molecular weight | 486.43 g/mol |
| Num. heavy atoms | 35 |
| Num. arom. heavy atoms | 23 |
| Fraction Csp3 | 0.12 |
| Num. rotatable bonds | 11 |
| Num. H-bond acceptors | 7 |
| Num. H-bond donors | 0 |
| Molar Refractivity | 120.75 |
| TPSA | 87.83 Å² |
| Lipophilicity | |
| Log *P*_o/w_ (iLOGP) | 0.00 |
| Log *P*_o/w_ (XLOGP3) | 4.44 |
| Log *P*_o/w_ (WLOGP) | 3.58 |
| Log *P*_o/w_ (MLOGP) | 2.27 |
| Log *P*_o/w_ (SILICOS-IT) | 1.51 |
| Consensus Log *P*_o/w_ | 2.36 |
| Water Solubility | |
| Log *S* (ESOL) | -5.41 |
| Solubility | 1.88e-03 mg/ml ; 3.86e-06 mol/l |
| Class | Moderately soluble |
| Log *S* (Ali) | -6.00 |
| Solubility | 4.83e-04 mg/ml ; 9.93e-07 mol/l |
| Class | Poorly soluble |
| Log *S* (SILICOS-IT) | -7.70 |
| Solubility | 9.75e-06 mg/ml ; 2.00e-08 mol/l |
| Class | Poorly soluble |
| Pharmacokinetics | |
| GI absorption | High |
| BBB permeant | No |
| P-gp substrate | Yes |
| CYP1A2 inhibitor | No |
| CYP2C19 inhibitor | Yes |
| CYP2C9 inhibitor | Yes |
| CYP2D6 inhibitor | No |
| CYP3A4 inhibitor | Yes |
| Log *K*_p_ (skin permeation) | -6.11 cm/s |
| Druglikeness | |
| Lipinski | Yes; 0 violation |
| Ghose | No; 1 violation: MW>480 |
| Veber | No; 1 violation: Rotors>10 |
| Egan | Yes |
| Muegge | Yes |
| Bioavailability Score | 0.55 |
| Medicinal Chemistry | |
|  |  |
| PAINS | 0 alert |
| Brenk | 1 alert: imine_1 |
| Leadlikeness | No; 3 violations: MW>350, Rotors>7, XLOGP3>3.5 |
| Synthetic accessibility | 4.12 |

|  |  |
| --- | --- |

**Compound 5**

| SMILES | [Na]OC(=O)[C@H](Cc1c[nH]cn1)/N=C/c1ccccc1OCc1nnn(c1)c1ccccc1 |
| --- | --- |

| Physicochemical Properties | |
| --- | --- |
| Formula | C_22_H_19_N_6_NaO_3_ |
| Molecular weight | 438.41 g/mol |
| Num. heavy atoms | 32 |
| Num. arom. heavy atoms | 22 |
| Fraction Csp3 | 0.14 |
| Num. rotatable bonds | 10 |
| Num. H-bond acceptors | 7 |
| Num. H-bond donors | 1 |
| Molar Refractivity | 112.27 |
| TPSA | 107.28 Å² |
| Lipophilicity | |
| Log *P*_o/w_ (iLOGP) | 0.00 |
| Log *P*_o/w_ (XLOGP3) | 2.66 |
| Log *P*_o/w_ (WLOGP) | 2.46 |
| Log *P*_o/w_ (MLOGP) | 1.03 |
| Log *P*_o/w_ (SILICOS-IT) | 2.03 |
| Consensus Log *P*_o/w_ | 1.63 |
| Water Solubility | |
| Log *S* (ESOL) | -4.08 |
| Solubility | 3.62e-02 mg/ml ; 8.27e-05 mol/l |
| Class | Moderately soluble |
| Log *S* (Ali) | -4.56 |
| Solubility | 1.20e-02 mg/ml ; 2.73e-05 mol/l |
| Class | Moderately soluble |
| Log *S* (SILICOS-IT) | -6.77 |
| Solubility | 7.45e-05 mg/ml ; 1.70e-07 mol/l |
| Class | Poorly soluble |
| Pharmacokinetics | |
| GI absorption | High |
| BBB permeant | No |
| P-gp substrate | Yes |
| CYP1A2 inhibitor | No |
| CYP2C19 inhibitor | Yes |
| CYP2C9 inhibitor | Yes |
| CYP2D6 inhibitor | No |
| CYP3A4 inhibitor | No |
| Log *K*_p_ (skin permeation) | -7.09 cm/s |
| Druglikeness | |
| Lipinski | Yes; 0 violation |
| Ghose | Yes |
| Veber | Yes |
| Egan | Yes |
| Muegge | Yes |
| Bioavailability Score | 0.55 |
| Medicinal Chemistry | |
|  |  |
| PAINS | 0 alert |
| Brenk | 1 alert: imine_1 |
| Leadlikeness | No; 2 violations: MW>350, Rotors>7 |
| Synthetic accessibility | 4.10 |

**Compound 6**

| SMILES | OC(=O)C(Cc1ccccc1)/N=C/c1ccccc1OCc1nnn(c1)c1ccccc1 |
| --- | --- |

| Physicochemical Properties | |
| --- | --- |
| Formula | C_25_H_22_N_4_O_3_ |
| Molecular weight | 426.47 g/mol |
| Num. heavy atoms | 32 |
| Num. arom. heavy atoms | 23 |
| Fraction Csp3 | 0.12 |
| Num. rotatable bonds | 9 |
| Num. H-bond acceptors | 6 |
| Num. H-bond donors | 1 |
| Molar Refractivity | 121.67 |
| TPSA | 89.60 Å² |
| Lipophilicity | |
| Log *P*_o/w_ (iLOGP) | 3.53 |
| Log *P*_o/w_ (XLOGP3) | 4.18 |
| Log *P*_o/w_ (WLOGP) | 3.81 |
| Log *P*_o/w_ (MLOGP) | 2.79 |
| Log *P*_o/w_ (SILICOS-IT) | 4.19 |
| Consensus Log *P*_o/w_ | 3.70 |
| Water Solubility | |
| Log *S* (ESOL) | -5.06 |
| Solubility | 3.75e-03 mg/ml ; 8.80e-06 mol/l |
| Class | Moderately soluble |
| Log *S* (Ali) | -5.77 |
| Solubility | 7.24e-04 mg/ml ; 1.70e-06 mol/l |
| Class | Moderately soluble |
| Log *S* (SILICOS-IT) | -7.55 |
| Solubility | 1.20e-05 mg/ml ; 2.81e-08 mol/l |
| Class | Poorly soluble |
| Pharmacokinetics | |
| GI absorption | High |
| BBB permeant | No |
| P-gp substrate | No |
| CYP1A2 inhibitor | No |
| CYP2C19 inhibitor | Yes |
| CYP2C9 inhibitor | Yes |
| CYP2D6 inhibitor | No |
| CYP3A4 inhibitor | Yes |
| Log *K*_p_ (skin permeation) | -5.93 cm/s |
| Druglikeness | |
| Lipinski | Yes; 0 violation |
| Ghose | Yes |
| Veber | Yes |
| Egan | Yes |
| Muegge | Yes |
| Bioavailability Score | 0.56 |
| Medicinal Chemistry | |
|  |  |
| PAINS | 0 alert |
| Brenk | 1 alert: imine_1 |
| Leadlikeness | No; 3 violations: MW>350, Rotors>7, XLOGP3>3.5 |
| Synthetic accessibility | 3.97 |

Top of Form

Bottom of Form

Top of Form

Bottom of Form

Top of Form

Bottom of Form

Top of Form

Bottom of Form
